# Phenotypic subgrouping and multi-omics analyses reveal reduced diazepam-binding inhibitor (DBI) protein levels in autism spectrum disorder with severe language impairment

**DOI:** 10.1371/journal.pone.0214198

**Published:** 2019-03-28

**Authors:** Chatravee Pichitpunpong, Surangrat Thongkorn, Songphon Kanlayaprasit, Wasana Yuwattana, Waluga Plaingam, Siriporn Sangsuthum, Wan Mohd Aizat, Syarul Nataqain Baharum, Tewin Tencomnao, Valerie Wailin Hu, Tewarit Sarachana

**Affiliations:** 1 M.Sc. Program in Clinical Biochemistry and Molecular Medicine, Department of Clinical Chemistry, Faculty of Allied Health Sciences, Chulalongkorn University, Bangkok, Thailand; 2 PhD Program in Clinical Biochemistry and Molecular Medicine, Department of Clinical Chemistry, Faculty of Allied Health Sciences, Chulalongkorn University, Bangkok, Thailand; 3 B.Sc. Program in Medical Technology, Faculty of Allied Health Sciences, Chulalongkorn University, Bangkok, Thailand; 4 College of Oriental Medicine, Rangsit University, Pathum Thani, Thailand; 5 Department of Clinical Chemistry, Faculty of Allied Health Sciences, Chulalongkorn University, Bangkok, Thailand; 6 Institute of Systems Biology (INBIOSIS), Universiti Kebangsaan Malaysia, Bangi, Selangor, Malaysia; 7 Age-related Inflammation and Degeneration Research Unit, Department of Clinical Chemistry, Faculty of Allied Health Sciences, Chulalongkorn University, Bangkok, Thailand; 8 Department of Biochemistry and Molecular Medicine, George Washington University School of Medicine and Health Sciences, Washington, DC, United States of America; Pacific Northwest National Laboratory, UNITED STATES

## Abstract

**Background:**

The mechanisms underlying autism spectrum disorder (ASD) remain unclear, and clinical biomarkers are not yet available for ASD. Differences in dysregulated proteins in ASD have shown little reproducibility, which is partly due to ASD heterogeneity. Recent studies have demonstrated that subgrouping ASD cases based on clinical phenotypes is useful for identifying candidate genes that are dysregulated in ASD subgroups. However, this strategy has not been employed in proteome profiling analyses to identify ASD biomarker proteins for specific subgroups.

**Methods:**

We therefore conducted a cluster analysis of the Autism Diagnostic Interview-Revised (ADI-R) scores from 85 individuals with ASD to predict subgroups and subsequently identified dysregulated genes by reanalyzing the transcriptome profiles of individuals with ASD and unaffected individuals. Proteome profiling of lymphoblastoid cell lines from these individuals was performed via 2D-gel electrophoresis, and then mass spectrometry. Disrupted proteins were identified and compared to the dysregulated transcripts and reported dysregulated proteins from previous proteome studies. Biological functions were predicted using the Ingenuity Pathway Analysis (IPA) program. Selected proteins were also analyzed by Western blotting.

**Results:**

The cluster analysis of ADI-R data revealed four ASD subgroups, including ASD with severe language impairment, and transcriptome profiling identified dysregulated genes in each subgroup. Screening via proteome analysis revealed 82 altered proteins in the ASD subgroup with severe language impairment. Eighteen of these proteins were further identified by nano-LC-MS/MS. Among these proteins, fourteen were predicted by IPA to be associated with neurological functions and inflammation. Among these proteins, diazepam-binding inhibitor (DBI) protein was confirmed by Western blot analysis to be expressed at significantly decreased levels in the ASD subgroup with severe language impairment, and the DBI expression levels were correlated with the scores of several ADI-R items.

**Conclusions:**

By subgrouping individuals with ASD based on clinical phenotypes, and then performing an integrated transcriptome-proteome analysis, we identified DBI as a novel candidate protein for ASD with severe language impairment. The mechanisms of this protein and its potential use as an ASD biomarker warrant further study.

## Introduction

Autism spectrum disorder (ASD) is a group of neurodevelopmental disorders thought to result from both genetic and environmental factors [1–4]. The Centers for Disease Control and Prevention (CDC) has reported that the prevalence of ASD is approximately 1 in 59 children in the United States [5]. According to the Diagnostic and Statistical Manual of Mental Disorders—Fifth Edition (DSM-5), ASD is characterized by (i) impairments in social interaction and communication and (ii) repetitive behaviors and restricted interests, without further subclassification into specific ASD subtypes [6]. However, there is a high degree of variation in ASD symptoms and severity. Some individuals with ASD exhibit severe language deficits, including a lack of verbal communication, whereas many others show little impairment in language and are capable of communication with others [7]. Such heterogeneity in the autism spectrum is thought to result from the combination of multiple molecular mechanisms and environmental factors, leading to the pathobiology specific to the clinical phenotypes of each ASD subpopulation. There is evidence that dividing individuals with ASD into subgroups is meaningful for identifying gene/protein candidates and molecular mechanisms that are uniquely associated with each ASD subpopulation and shared among subpopulations, which may be useful for ASD diagnosis and/or treatment in the future [8–17]. However, clinical biomarkers are not available for ASD diagnosis or the specific differentiation of severity. Accurate predictive clinical biomarkers for the diagnosis and confirmation of the disorder, as well as for ASD subtyping, are still needed.

Several studies involving transcriptome profiling analysis of lymphoblastoid cell lines (LCLs) derived from patients with ASD and unaffected controls have revealed a number of transcripts that are differentially expressed in this condition [15–18]. Interestingly, these transcripts include genes that are known to be associated with neurological functions that are negatively impacted in ASD. The protein products encoded by some of the differentially expressed genes found in LCLs, including *RORA* and *BCL2*, also show reduced expression in the brains of individuals with ASD [14]. In addition to coding transcriptome profiling, several studies have investigated non-coding RNA transcripts in LCLs [19,20]. Among the dysregulated miRNAs in LCLs, miR-23a and miR-106b were reported to exhibit altered expression by Abu-Elneel *et al*. (2008) [21], who studied postmortem cerebellar tissue from patients with autism, suggesting that altered levels of certain transcripts in LCLs may reflect the molecular pathological condition of ASD in the brain. These data support the use of LCLs as a surrogate cell population for studying ASD. The levels of certain transcripts, such as *RORA*, were found to correlate with the levels of their protein products and could provide insights into important molecular mechanisms associated with ASD [13,22–24]. However, most of the differentially expressed transcripts related to ASD show little correlation with the levels of their encoded proteins, which is likely due to post-transcriptional regulatory processes. Moreover, differences in the genes and transcripts associated with ASD have shown little or no reproducibility in different cohorts, which is partly due to the high degree of heterogeneity in the autism spectrum.

Several studies have therefore conducted proteome analyses of ASD samples using various tissue types, including postmortem brain tissues [25], serum [26–28], plasma [29–31], lymphocytes [32], neonatal blood [33,34], urine [35], and saliva [36–38]. Broek et al. (2014) conducted targeted selected reaction monitoring mass spectrometry (SRM-MS) analysis of postmortem brain tissues from the prefrontal cortex and cerebellum of individuals with ASD and matched controls [25]. Several proteins associated with myelination, synaptic vesicle regulation, and energy metabolism have been reported to be dysregulated in ASD brain tissues. In addition, in search of potential biomarkers, several studies have interrogated proteome profiles in the peripheral blood of patients with ASD. Corbett et al. (2007) performed liquid chromatography-electrospray ionization-mass spectrometry (LC-ESI-MS) with time-of-flight (TOF) to identify differentially expressed peptides in the serum of children with ASD compared to typically developing children [26]. These authors reported that apolipoprotein (apo) B-100, complement factor H-related protein (FHR1), complement C1q, and fibronectin 1 (FN1) were dysregulated in serum from individuals with ASD. Moreover, Ngounou et al. (2014) performed a proteomic analysis of serum from individuals with ASD and unaffected controls using Tricine-PAGE followed by LC-MS/MS analysis [28]. ApoA1 and ApoA4, which are involved in cholesterol metabolism, and PON1, which is involved in detoxification, were found to be significantly differentially expressed between the groups. A multiplex immunoassay profiling analysis of serum samples from 37 individuals with a diagnosis of ASD and their matched, nonaffected siblings revealed age-dependent differences in the levels of 12 proteins involved in inflammation, growth, and hormonal signaling, emphasizing the importance of subgrouping and the analysis of samples by age in ASD proteomic studies [27]. Cortelazzo et al. (2016) analyzed plasma proteins in 30 subjects with ASD and 30 individuals with typical development [30]. A total of 12 proteins were found to be dysregulated, with 10 being associated with the acute inflammatory response. Moreover, cytokine profiling analysis of neonatal blood spots from children with ASD revealed that interleukin (IL)-1β and IL-4 were associated with ASD [34]. Interestingly, these relationships were reported to vary by ASD symptom severity. Elevated IL-4 was associated with increased odds of severe ASD, whereas IL-1β was associated with increased odds of mild/moderate ASD, suggesting that subgrouping ASD based on symptom severity might be useful for identifying blood-based specific markers for ASD subpopulations. Although several proteomic studies can be found in the literature, one of the challenges of ASD proteomic research is that the levels of candidate proteins seem to show little or no reproducibility in independent cohorts. This critical issue, which impedes the understanding of ASD proteome biology and the identification of protein markers, is the result of (i) technical issues, including different levels of proteome coverage and varying information provided by proteomic platforms, and (ii) biological complexities, such as the heterogeneity of the autism spectrum and different tissue types.

In the present study, we therefore sought to investigate the proteome profiles of patients with ASD by reducing the heterogeneity of the ASD population using a phenotypic subgrouping strategy that we employed in recent studies [10,16,17,39–41], followed by transcriptome and proteome profiling analyses. First, a cluster analysis of clinical phenotypes obtained from the Autism Diagnostic Interview-Revised (ADI-R) scores from individuals with ASD was performed to identify subgroups/clusters of ASD based on clinical phenotypes. Transcriptome profile data of LCLs from these individuals and age-/sex-matched unaffected individuals were obtained from the NCBI GEO DataSets database and reanalyzed to identify differentially expressed genes in both the ASD population as well as in each ASD subgroup. Moreover, proteome profiling analysis of LCLs from a subgroup of patients with ASD with severe language impairment and age-/sex-matched unaffected individuals was conducted. These data were compared to lists of differentially expressed transcripts as well as significant results from previous proteomic studies. Biological functions and pathways related to these proteins were predicted, and selected proteins were further validated via a Western blot analysis and correlated with the scores of ADI-R items.

## Materials and methods

### Collection of ADI-R data and identification of phenotypic subgroups

Scores from a total of 123 items in ADI-R structured interviews of 85 male individuals with ASD were obtained from a previously published study by Hu and Steinberg (2009) [41]. These ADI-R score sheets were downloaded from the Autism Genetic Research Exchange (AGRE) phenotype database [42], and the individuals were selected based on previously described criteria [16]. Briefly, all individuals with ASD were males diagnosed through an ADI-R diagnostic interview. Individuals with cognitive impairment (Raven's scores <70), those with known genetic or chromosomal abnormalities (e.g., Fragile X, Rett syndrome, tuberous sclerosis, chromosome 15q11–q13 duplication), those born prematurely (<35 weeks gestation), and those with diagnosed comorbid psychiatric disorders (e.g., bipolar disorder, obsessive compulsive disorder, severe anxiety) were excluded. Impairment of spoken language was additionally confirmed based on low standard scores (<80) in the Peabody Picture Vocabulary Test.

LCLs from these individuals with ASD (n = 85) and sex-/age-matched unaffected individuals (n = 29) have been subjected to transcriptome profiling analysis [16], and the transcriptome profile data of the LCLs from these individuals have been deposited in the NCBI GEO DataSets repository [43] (GSE15402). The majority of the controls (24 out of 29) were unrelated to the cases, whereas 5 were unaffected siblings. The demographic information for all individuals examined in this study is shown in **S1 Table**.

Unsupervised hierarchical clustering (HCL) analysis and principal component analysis (PCA) of the ADI-R data were performed using Multiple Experiment Viewer (MeV) (http://mev.tm4.org; [44]) as previously described [41] to identify phenotypic subgroups based on clustering patterns. The use of lymphoblastoid cell lines in this study was reviewed by the GWU Office of Human Research and determined to be “Exempt” from full IRB review for human subject research because all of the cell lines and phenotypic data used in this study were deidentified with respect to the donor. Moreover, there were never any direct interactions with any of the individuals whose cells were employed in this study.

### Reanalysis of transcriptome profile data

To identify differentially expressed genes in each ASD phenotypic subgroup, the transcriptome profiles of LCLs derived from individuals with ASD and sex-/age-matched unaffected individuals were downloaded from the gene expression profiling results of Hu et al. (2009) (GSE number: GSE15402) in the NCBI GEO DataSets database [16]. In addition to the GSE15402 study, we obtained transcriptome data from four other gene expression profiling studies involving peripheral blood cells or cell lines derived from peripheral blood cells from the NCBI GEO DataSets database (GSE25507, GSE6575, GSE18123, and GSE42133) [45–48]. Differentially expressed transcripts from each study were then identified, as described in previous reports [8,9]. The normalized transcriptome data were uploaded to the Multiple Experiment Viewer (MeV) program [44], and a 70% data cutoff filter was applied to remove genes for which log_2_ intensity values were missing in >30% of the samples in each study. Significantly differentially expressed genes were identified using t-tests with standard Bonferroni correction (P-value < 0.05).

### Collection of published proteome profile data

We searched the NCBI PubMed database to obtain a list of significantly differentially expressed proteins in ASD that have been published in the literature. A total of 14 proteomic studies in the NCBI PubMed database were used [25–38]. All of the ASD proteome studies were published from 2007–2017 and employed human tissue samples, including postmortem brain tissues, serum, plasma, neonatal blood, urine, and saliva. The significant protein hits identified and reported by all proteome studies were obtained and combined.

### Cell culture

Lymphoblastoid cell lines derived from the peripheral lymphocytes of individuals with ASD (n = 36) and sex-/age-matched unaffected individuals (n = 20) who were also included in the transcriptome study GSE15402 were obtained from the Autism Genetics Resource Exchange Repository (AGRE, Los Angeles, CA, USA). The demographic information and phenotypic cluster for all individuals whose LCLs were employed for cell culture are shown in **S1 Table**. All of the LCLs were cultured in Hyclone RPMI-1640 (GE Healthcare, Chicago, IL, USA) supplemented with 15% fetal bovine serum (Sigma-Aldrich, St. Louis, MO, USA) and 1% penicillin-streptomycin-amphotericin B solution (Mediatech, Manassas, VA, USA) according to the protocol of the Rutgers University Cell and DNA Repository, as previously described [15]. Briefly, cell culture was performed in a humidified atmosphere of 5% CO_2_ at 37°C. The cells were split 1:2 every 3−4 days and were harvested for protein isolation 3 days after splitting, when the cells were in the logarithmic growth phase. The cells were then pelleted and used for subsequent protein isolation. In addition to proteins employed for this study, we also obtained DNA and RNA from the same samples for future investigation. To allow serial isolation of DNA, RNA, and proteins from the same samples, the cells were therefore resuspended in RNAlater solution (Thermo Fisher Scientific, Waltham, MA, USA) and stored at -80°C until DNA/RNA/protein isolation. According to the manufacturer’s protocol, RNAlater solution can be used for stabilizing DNA, RNA, and proteins in cell samples.

### Protein isolation and purification

Proteins were extracted from LCLs using the GENEzol reagent (Geneaid Biotech Ltd., New Taipei City, Taiwan) according to the manufacturer’s protocol, which also allowed for the isolation of DNA and RNA for future study. Briefly, LCLs stored in RNAlater were centrifuged to pellet the cells. RNAlater was then removed, and the GENEzol reagent was added to the cell pellet to lyse the cells. A total of 200μL of chloroform was added to the GENEzol extract, followed by centrifugation. The aqueous phase was then removed, and 100% ethanol was added to the interphase and the organic phase. After centrifugation to pellet the DNA, the supernatant was transferred to a new tube for protein isolation. To precipitate proteins, isopropanol was added to the phenol-ethanol supernatant, followed by centrifugation for 10 minutes at 12,000 g at 4°C. The protein pellet was washed 3 times in a wash solution consisting of 0.3 M guanidine hydrochloride in 95% ethanol and then washed once in 100% ethanol. The protein pellet was subsequently air dried and resuspended in 200 μl of a lysis buffer consisting of 7 M urea, 2 M thiourea, 4% w/v CHAPS, and 100 mM dithiothreitol (DTT). The concentrations of the protein samples were measured using Bradford protein assays, with bovine serum albumin (BSA) as a standard. A 5μg sample of each protein extract was separated via 12% SDS-PAGE and stained with a colloidal Coomassie Brilliant Blue G-250 solution consisting of ammonium sulfate, 85% phosphoric acid, Coomassie Brilliant Blue G-250, and methanol to assess the integrity of the proteins prior to two-dimensional gel electrophoresis.

### Two-dimensional gel electrophoresis

To screen for proteins differentially expressed in ASD with severe language impairment, two-dimensional gel electrophoresis was conducted using protein samples from LCLs derived from individuals with ASD with severe language impairment (n = 6) or sex-/age-matched unaffected individuals (n = 6). A total of 200 μg of purified protein was loaded onto Immobiline DryStrips (pH 3–10 L, 13 cm) with rehydration buffer consisting of 7 M urea, 2 M thiourea, 4% CHAPS, 100 mM DTT, 2% Biolyte, and 0.5% bromophenol blue. The strips were rehydrated at 20°C for 12 h. The proteins were subjected to isoelectric focusing with a Multiphor III Electrophoresis System (Amersham Biosciences, Little Chalfont, UK) at the following voltages: 500 V for 300 Vh (step and hold), 1000 V for 800 Vh (gradient), 8000 V for 11,300 Vh (gradient), and 8000 V for 4400 Vh (step and hold). After focusing, the immobilized pH gradient (IPG) strips were reduced in equilibration buffer consisting of 50 mM Tris-HCl (pH 8.8), 6 M urea, 30% v/v glycerol, 2% SDS w/v, and 1% bromophenol blue with 100 mM DTT for 30 minutes. The strips were then alkylated in equilibration buffer containing 150 mM iodoacetamide (IAA) for 45 minutes. The proteins were separated in the second dimension via 12.5% SDS-PAGE. Each pooled sample was run in triplicate 2D gels. The gels were stained with Coomassie Brilliant Blue G250 overnight and destained with Milli-Q water until the background color was clear. The 2D gels were scanned at a 600 μm/pixel resolution and analyzed with ImageMaster 2D Platinum version 7.0 software (Amersham Biosciences). Each sample was run in triplicate, and differentially expressed protein spots were detected as significant by ANOVA with a P-value of less than 0.05.

### Tryptic in-gel digestion and protein identification by LC-MS/MS

The significantly differentially expressed protein spots were excised from the gels and destained with 50% acetonitrile (ACN) in 100 μL of 25 mM ammonium bicarbonate. After destaining, tryptic digestion was performed according to a previously described method [49]. The resulting peptide mixtures were analyzed with an UltiMate 3000 RSLCnano System (Ultimate 3000, Dionex, USA) coupled to a micrOTOF-Q II ESI-Qq-TOF mass spectrometer (Bruker Daltonics, Germany). The MS/MS spectra data from each sample were searched against the NCBI databases using the MASCOT search engine (Matrix Science, London, UK) (http://www.matrixscience.com/) [50].

### Prediction of biological functions and networks

The biological functions and networks associated with the differentially expressed proteins were predicted using Ingenuity Pathway Analysis (IPA) version 6.0 (QIAGEN Bioinformatics, https://www.qiagenbioinformatics.com/products/ingenuity-pathway-analysis/). IPA is a manually curated web-based functional analysis and knowledge discovery tool for omic data. IPA Knowledgebase collects from the literature all information about diseases, canonical pathways, biological functions, interactions, and regulators associated with genes and proteins in any context. Thus, a single gene/protein may have been reported to be involved in several different categories based on previously published research. To determine the association between a list of genes/proteins and each disease/process/pathway, IPA overlaps the list of genes/proteins provided by the user with the list of genes/proteins associated with a category in the database. The P-value is then calculated using the Fisher Exact test as a measure of the likelihood that the association between a set of focus genes in the experiment and a given disease/process/pathway is due to random chance. A P-value < 0.05 is considered to be a statistically significant, nonrandom association. The GeneCards database was also employed to predict functions and phenotypes associated with proteins (www.genecards.org) [51]. Moreover, to determine whether the differentially expressed proteins had previously been associated with ASD or reported in ASD studies as “ASD candidate” genes/proteins, the list of differentially expressed proteins was searched against AutismKB Knowledgebase (http://autismkb.cbi.pku.edu.cn/) [52].

### Western blot analysis

Western blot analysis was performed to validate selected differentially expressed proteins in the ASD phenotypic subgroup with severe language impairment identified by 2D gel electrophoresis and subsequent mass spectrometry analysis. A total of 15 μg of each LCL protein sample was separated via 10% SDS-PAGE and transferred to a 0.2 μM Immun-Blot PVDF membrane (Bio-Rad Laboratories, Hercules, CA, USA) in a Mini-PROTEAN Tetra system. The membranes were then blocked with 5% nonfat dry milk (Vivantis Technologies Sdn. Bhd., Malaysia) in TBST for 1 h at room temperature. After blocking, the membranes were incubated overnight with a 1:8,000 dilution of rabbit monoclonal anti-human IDH2 antibody [EPR7576] (ab129180, Abcam) or a 1:12,000 dilution of rabbit polyclonal anti-human DBI antibody (ab196485, Abcam), followed by incubation in a 1:12,000 dilution of preadsorbed donkey anti-rabbit IgG H&L (HRP) (ab7083, Abcam). As a protein loading control, the membranes were stripped with 0.2 M NaOH for 5 minutes and reprobed overnight with a 1:8,000 dilution of a rabbit polyclonal anti-GAPDH antibody (sc25778, Santa Cruz Biotechnology, Dallas, Texas, USA). All of the membranes were visualized with Amersham ECL Select Western Blotting Detection Reagent with ECL Hyperfilm. The band images were obtained with the Syngene Gbox EF Gel Documentation System using the GeneTools program. The significance of differences between the groups was calculated using two-tailed paired t-test analysis. Pearson correlation analysis of the levels of selected proteins and ADI-R scores was conducted using IBM SPSS Statistics version 22.

## Results

**Fig 1:**
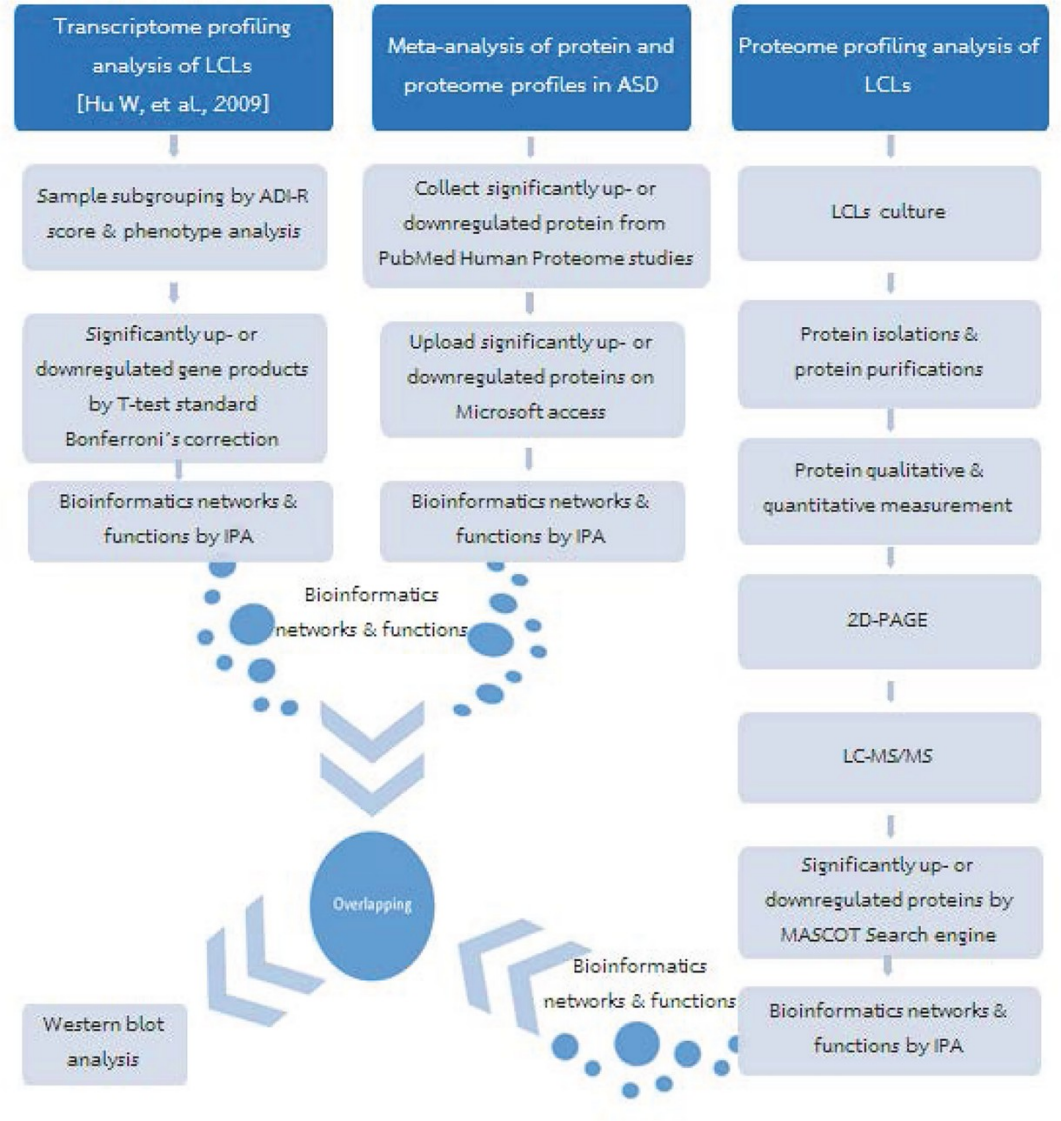
Schematic diagram showing the experimental workflow of this study.

**Fig 2:**
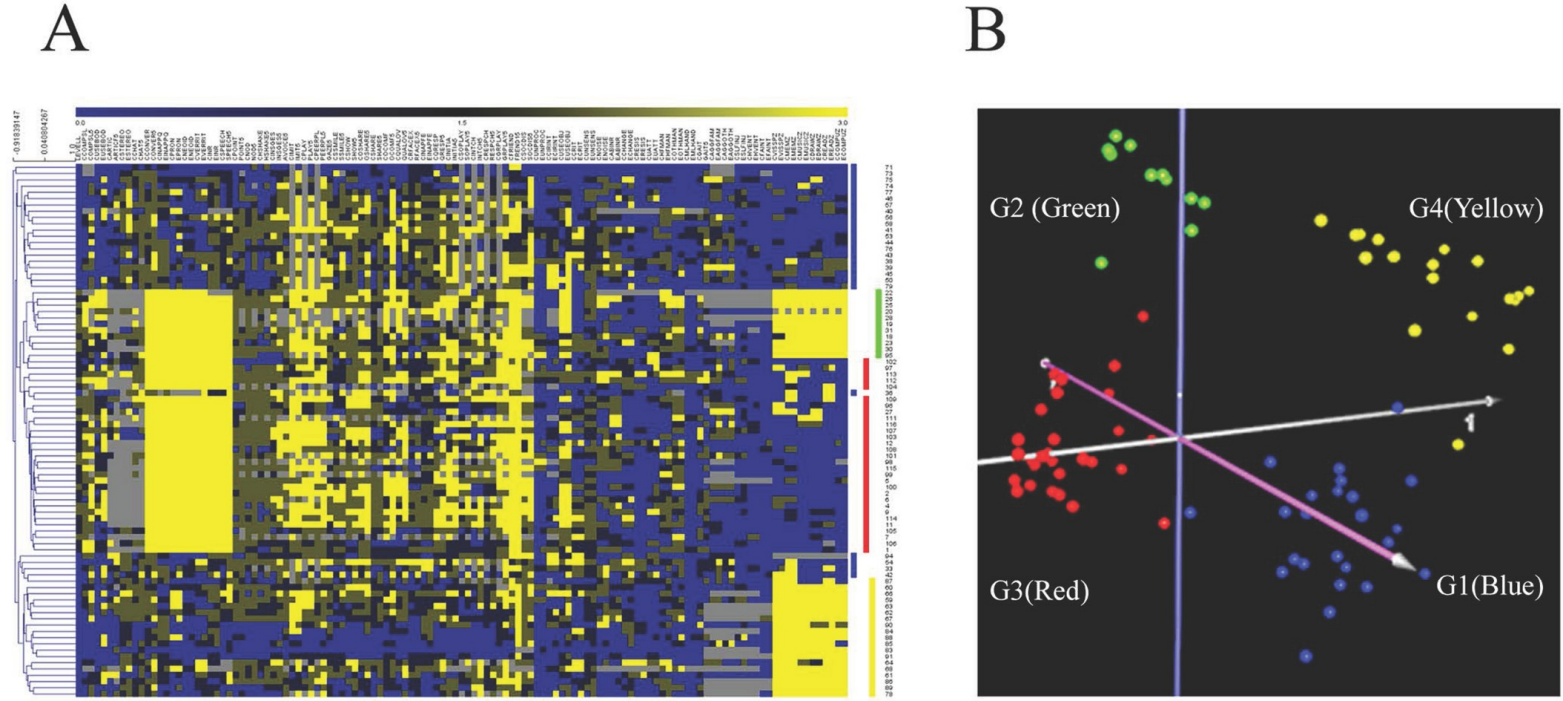
Cluster analysis of ADI-R scores of individuals with ASD. (A) Hierarchical cluster analysis of 123 ADI-R scores of 85 individuals with ASD. Each column in the heat map represents each ADI-R question, whereas each row represents each individual with ASD. The colors in the heat map represent ADI-R scores and reflect the severity of that behavior/function, ranging from a lower score (blue, less severe) to a higher score (yellow, more severe). (B) Principal component analysis (PCA) of the ADI-R scores of 85 individuals with ASD used in this study revealed four distinct clusters (G1–G4). Each dot represents an individual with ASD.

The experimental workflow of this study is illustrated in **Fig 1**. First, to reduce the heterogeneity of the ASD population prior to subsequent transcriptome and proteome profiling analyses, we performed a cluster analysis of clinical phenotypes associated with ASD. For this analysis, we obtained the scores from 123 items in the ADI-R questionnaire and the demographic information of 85 individuals with ASD from a study by Hu & Steinberg published in 2009 [41]. These 123 ADI-R items cover a broad spectrum of behaviors and functions across multiple ASD domains, including language, nonverbal communication, social interactions, play skills, interests and behaviors, physical sensitivities and mannerisms, aggression, and savant skills, as previously described [41]. Unsupervised hierarchical clustering (HCL) and principal component analysis (PCA) of the ADI-R score data were performed (**Fig 2**). The HCL and PCA of the ADI-R scores showed that the individuals with ASD separated distinctly into 4 main clusters, labeled G1 (blue, n = 24 individuals), G2 (green, n = 11 individuals), G3 (red, n = 30 individuals), and G4 (yellow, n = 20 individuals), as shown in **Fig 2**. Consistent with a previous study by Hu and Steinberg (2009), all of the individuals in each subgroup appeared to share a similar pattern of clinical phenotypes. Interestingly, individuals in G3 (red) exhibited scores for ADI-R items related to language that were higher than in the other groups, reflecting a higher degree of severity of language impairment.

### Transcriptome profiling analysis of LCLs

**Fig 3:**
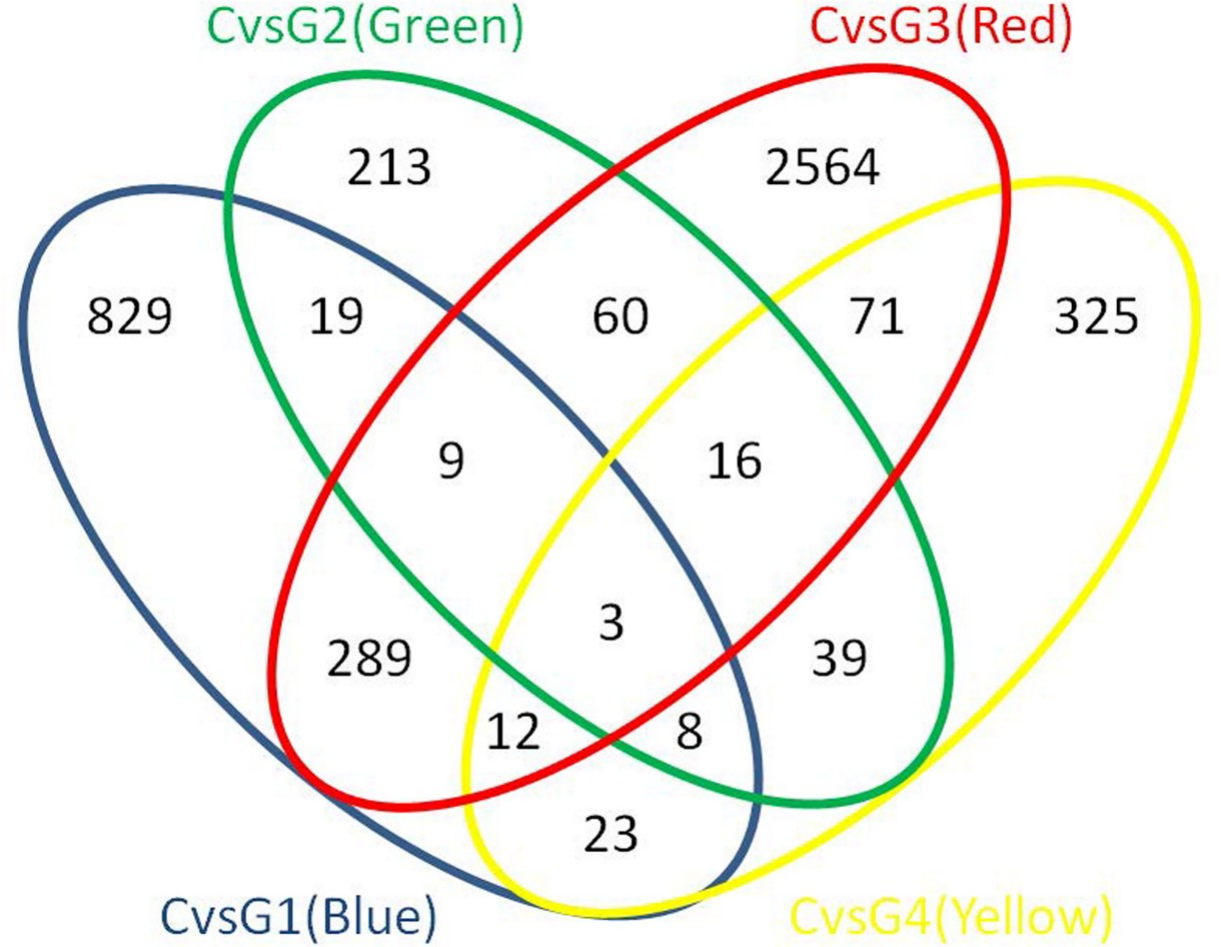
Overlap analysis of differentially expressed transcripts in the ASD phenotypic subgroups, as determined by the cluster analysis of ADI-R scores. The Venn diagram shows differentially expressed transcripts across the identified ASD subtypes, with significance determined using t-tests with standard Bonferroni correction (P-value < 0.05).

To identify differentially expressed transcripts in these 85 individuals with ASD and in each of the four ASD phenotypic subgroups identified by the cluster analysis, the transcriptome profiles of LCLs derived from these 85 individuals with ASD and 29 sex-/age-matched unaffected individuals were obtained from the NCBI GEO DataSets database (GSE15402) and reanalyzed. To ensure that the expression fold-change determinations for a particular gene/transcript were based on a majority of the samples tested, a 70% cutoff filter was applied. As many as 14,838 out of 41,472 probes were abundantly expressed in at least 70% of samples, which reflected substantial abundance of transcripts across LCL samples for the subsequent significance analysis of differentially expressed transcripts and also supported the feasibility of using LCLs for gene expression profiling analysis. Significantly differentially expressed transcripts in ASD and in each ASD phenotypic subgroup were identified via t-test analysis with standard Bonferroni correction (P-value < 0.05). The numbers of significantly differentially expressed transcripts and the corresponding proteins in the all-ASD group and in each subgroup are shown in **Table 1**. When all of the individuals with ASD were combined into one group, as many as 815 transcripts encoding 384 proteins were found to be dysregulated in ASD. After the ASD cases were further subgrouped via the ADI-R cluster analysis, we found that the G3 (red) subgroup exhibited the highest number of differentially expressed transcripts, including 3,024 transcripts corresponding to 1,726 proteins. The lists of differentially expressed transcripts and corresponding proteins in the all-ASD group and in each subgroup are shown in **S2 Table**. An overlap analysis of these differentially expressed transcripts revealed 549 transcripts that were altered in more than one subgroup as well as transcripts that were disrupted in only a specific subgroup (**Fig 3**). We next used IPA to analyze the biological functions of these differentially expressed transcripts. We found that the significantly differentially expressed transcripts in every subgroup were significantly associated with the “developmental disorder” and “neurological disease” categories (P-value < 0.05). Interestingly, we found that the differentially expressed transcripts in G3 (red) were significantly associated with “autism or intellectual disability” (n = 59 genes, P-value = 0.008), “mental retardation” (n = 56 genes, P-value = 0.006), and “congenital malformation of the brain” (n = 28 genes, P-value = 0.016). The results of this gene ontology analysis are shown in **S3 Table**. Compared to the other subgroups, the G3 (red) subgroup exhibited the greatest number of differentially expressed transcripts, suggesting that this subgroup might have consisted of individuals with the most severe form of ASD. Moreover, while all of the lists of differentially expressed transcripts from all phenotypic subgroups were significantly associated with ASD-related neurological functions or comorbid disorders, it is notable that the list of differentially expressed transcripts in the G3 (red) subgroup was the only list that was significantly associated with “autism” by IPA. We therefore focused on the G3 (red) subgroup in the subsequent proteome profiling analysis.

**Table 1:** Numbers of differentially expressed transcripts and corresponding protein products in different comparisons.

Comparison	#Differentially Expressed Transcripts	#Corresponding Protein Products
Control vs All ASD	815	384
Control vs G1 (Blue) subgroup	1,192	643
Control vs G2 (Green) subgroup	367	164
Control vs G3 (Red) subgroup	3,024	1,726
Control vs G4 (Yellow) subgroup	497	243

A total of 815 differentially expressed transcripts were identified by a 2-class t-test analysis with standard Bonferroni correction, comparing the gene expression between controls and all ASD individuals without subgrouping. Then, ASD individuals were further subgrouped, and the differentially expressed transcripts were identified by 2-class t-test analyses with standard Bonferroni correction for each of the ASD subgroups and controls.

### Proteome profiling analysis of ASD

**Fig 4:**
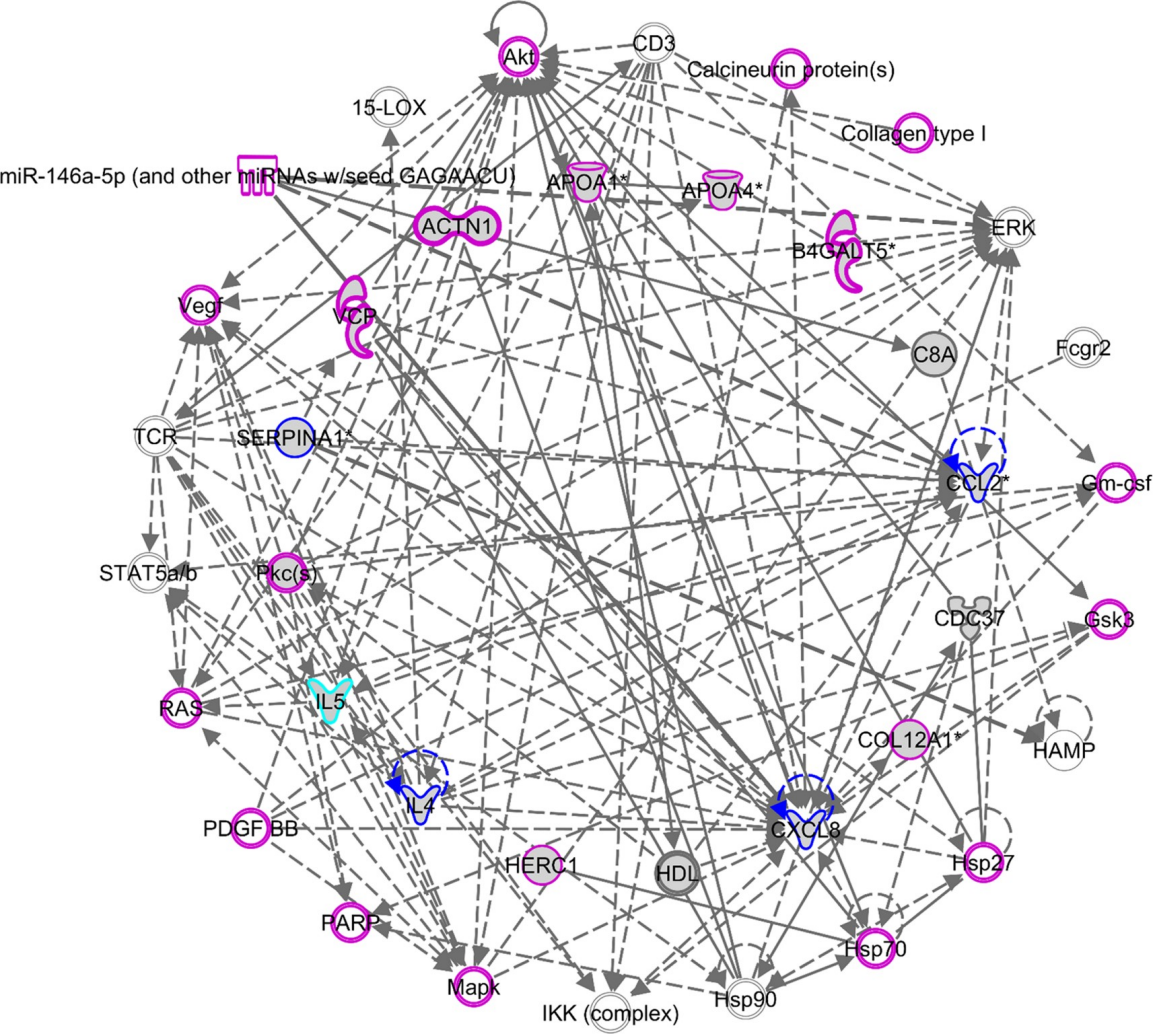
Biological network of differentially expressed proteins identified by previous ASD proteome studies. The list of 145 differentially expressed proteins from previous ASD proteome studies was analyzed, and a protein-protein interaction network was created using IPA. One of the networks is shown in this figure. Several proteins in the network were significantly associated with developmental disease (highlighted in purple), neurological disease (highlighted in light blue), and both developmental disease and neurological disease (highlighted in blue).

To determine whether the differentially expressed transcripts in LCLs reflected dysregulation at the proteome level, we conducted a hypergeometric distribution analysis between the list of differentially expressed transcripts in the all-ASD group and the list of differentially expressed proteins identified by previously published proteomic analyses of ASD. For this analysis, we employed the list of 815 differentially expressed transcripts in the all-ASD group that corresponded to 384 proteins (**S2 Table**). Notably, the number of predicted protein products was lower than the number of transcripts because multiple transcripts encode the same protein. Moreover, several transcripts in the original transcriptome dataset encode hypothetical proteins, potential noncoding RNAs, or unidentified probes. For the list of proteins, we collected data on significantly differentially expressed proteins from 14 proteomic studies in the NCBI PubMed database [25–38]. The details of all of the selected studies as well as the numbers of differentially expressed proteins identified in each study are shown in **Table 2**. A total of 145 proteins were identified as differentially expressed in at least one ASD proteomic study. Ontology analysis of these proteins using IPA revealed that these proteins were associated with the “neurological disease” (n = 86 proteins, P-value = 7.46E-23–3.41E-08), “psychological disorder” (n = 66 proteins, P-value = 7.46E-23–6.01E-08), and “embryonic development” (n = 9 proteins, P-value = 4.62E-08–4.62E-08) categories. Moreover, these proteins were associated with known ASD-related functions, including “inflammatory disease” (n = 71 proteins, P-value = 9.06E-26–1.55E-07), “lipid metabolism” (n = 31 proteins, P-value = 1.86E-22–1.47E-07), “free radical scavenging” (n = 26 proteins, P-value = 8.58E-20–2.36E-09), and “digestive system development and function” (n = 18 proteins, P-value = 3.27E-15–6.15E-12) [53–56]. A biological network of these proteins is shown in **Fig 4**.

**Table 2:** Previously published ASD proteome profiling studies with data used in this study and the number of significant proteins identified by each study.

Year	Study Title	Sample Type	# Differently Expressed Proteins
**2007**	A proteomic study of serum from children with autism showing differential expression of apolipoproteins and complement proteins [26]	Serum	5
**2008**	Hypo-Phosphorylation of Salivary Peptidome as a Clue to the Molecular Pathogenesis of Autism Spectrum Disorders [38]	Saliva	8
**2011**	A Proteomic Investigation of B Lymphocytes in an Autistic Family: A Pilot Study of Exposure to Natural Rubber Latex (NRL) May Lead to Autism [32]	Lymphocytes	14
**2013**	Identification of an age-dependent biomarker signature in children and adolescents with autism spectrum disorders [27]	Serum	12
**2014**	Neonatal cytokines and chemokines and risk of Autism Spectrum Disorder: the Early Markers for Autism (EMA) study: a case-control study [33]	Neonatal blood spots	2
**2014**	Proteomic analysis of postmortem brain tissue from autism patients: evidence for opposite changes in prefrontal cortex and cerebellum in synaptic connectivity-related proteins [25]	Postmortem brain	13
**2014**	A pilot proteomic study of protein markers in autism spectrum disorder [28]	Serum	3
**2015**	Comparative two-dimensional polyacrylamide gel electrophoresis of the salivary proteome of children with autism spectrum disorder [36]	Saliva	40
**2015**	A Pilot Proteomic Analysis of Salivary Biomarkers in Autism Spectrum Disorder [37]	Saliva	18
**2015**	Neonatal Cytokine Profiles Associated with Autism Spectrum Disorder [34]	Neonatal blood spots	16
**2016**	Urine Protein Biomarker Candidates for Autism [35]	Urine	25
**2016**	Expression and oxidative modifications of plasma proteins in autism spectrum disorders: Interplay between inflammatory response and lipid peroxidation [30]	Plasma	13
**2017**	Redox proteomic identification of carbonylated proteins in autism plasma: insight into oxidative stress [29]	Plasma	2
**2017**	iTRAQ-based proteomic analysis reveals protein profile in plasma from children with autism [31]	Plasma	24

**Fig 5:**
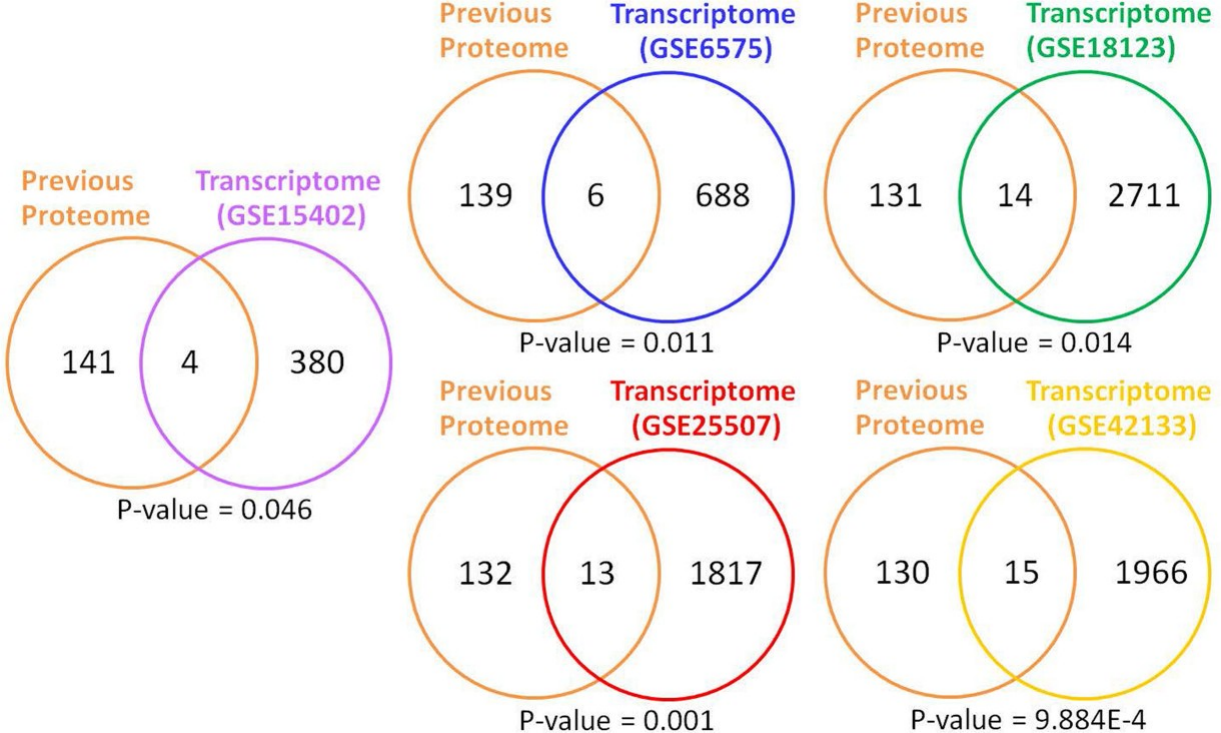
Overlap analysis between the list of differentially expressed proteins from previous ASD proteomic studies and the list of proteins encoded by differentially expressed transcripts from ASD transcriptome studies. P-values were calculated via hypergeometric distribution analysis.

A hypergeometric distribution analysis between the list of 145 differentially expressed proteins identified in previous ASD proteomic analyses and the 384 proteins encoded by the differentially expressed transcripts in the all-ASD group revealed a significant association (P-value < 0.05) between the transcriptome and proteome data, with 4 proteins in common (**Fig 5**). Moreover, the list of 3,024 differentially expressed transcripts in the G3 (red) subgroup that corresponded to 1,726 proteins (**S2 Table**) was also used for comparison. A total of 8 proteins were found in common.

In addition to the LCL transcriptome dataset (GSE15402), we compared the list of 145 differentially expressed proteins from previous ASD proteomic analyses to the lists of predicted proteins identified by four other ASD transcriptome studies (GSE6575, GSE18123, GSE25507, and GSE42133) involving peripheral blood cells or whole blood (**Fig 5**). Consistent with the LCL transcriptome dataset, there was a significant association between differentially expressed proteins in ASD from previous proteome studies and the lists of predicted proteins from all four ASD transcriptome studies. Among the proteins encoded by the transcripts that were differentially expressed in ASD LCLs or blood, several have been reported to be dysregulated in ASD serum/plasma or blood based on proteome studies (**Table 3**). These findings suggest that the dysregulation of at least some transcripts in LCLs or peripheral blood cells might reflect molecular changes at the proteome level.

**Table 3:** Hypergeometric distribution analysis between the list of differentially expressed proteins from previous ASD proteomic studies and the list of proteins encoded by differentially expressed transcripts from ASD transcriptomic studies using LCLs, peripheral blood cells, or whole blood.

Comparison	Transcriptome GEO Datasets	P-values	# Overlapping Proteins	Protein Symbols
ASD Proteome (all sources)	GSE15402	**0.047**	4	**PPP1R2**, **FGB**, PRH1, C5
	GSE6575	**0.011**	6	PRPF4, PCMTD1, **CDC37**, **ENO1**, MBP, **VCP**
	GSE18123	**0.014**	14	ATP6V1C1, B2M, ITGA6, HERC1, **TYK2**, **THBS1**, PCMTD1, MBP, **ACTN1**, **C1QC**, **PRKCD**, **EIF4G1**, **ITGA2B**, **MAP2K5**
	GSE25507	**0.001**	13	**MAP4**, A2M, MXRA8, **CCL4**, GRTP1, STATH, **CDC27**, STX1A, **IGHA1**, PIP, **APOA1**, **ACTN1**, SELENBP1
	GSE42133	**9.88E-04**	15	**TLN1**, B2M, **SERPINA4**, VAC14, PCMTD1, MBP, **ACTG1**, ANXA1, **SERPINA1**, AMY2A, **PPP1R2**, PRPF4, CTSL, HTRA2, PIP

P-values were calculated via hypergeometric distribution analysis. The proteins in bold are those encoded by the differentially expressed transcripts that were also differentially expressed in blood from individuals with ASD.

#### Proteome profiling analysis of LCLs from patients with ASD with severe language impairment

**Fig 6:**
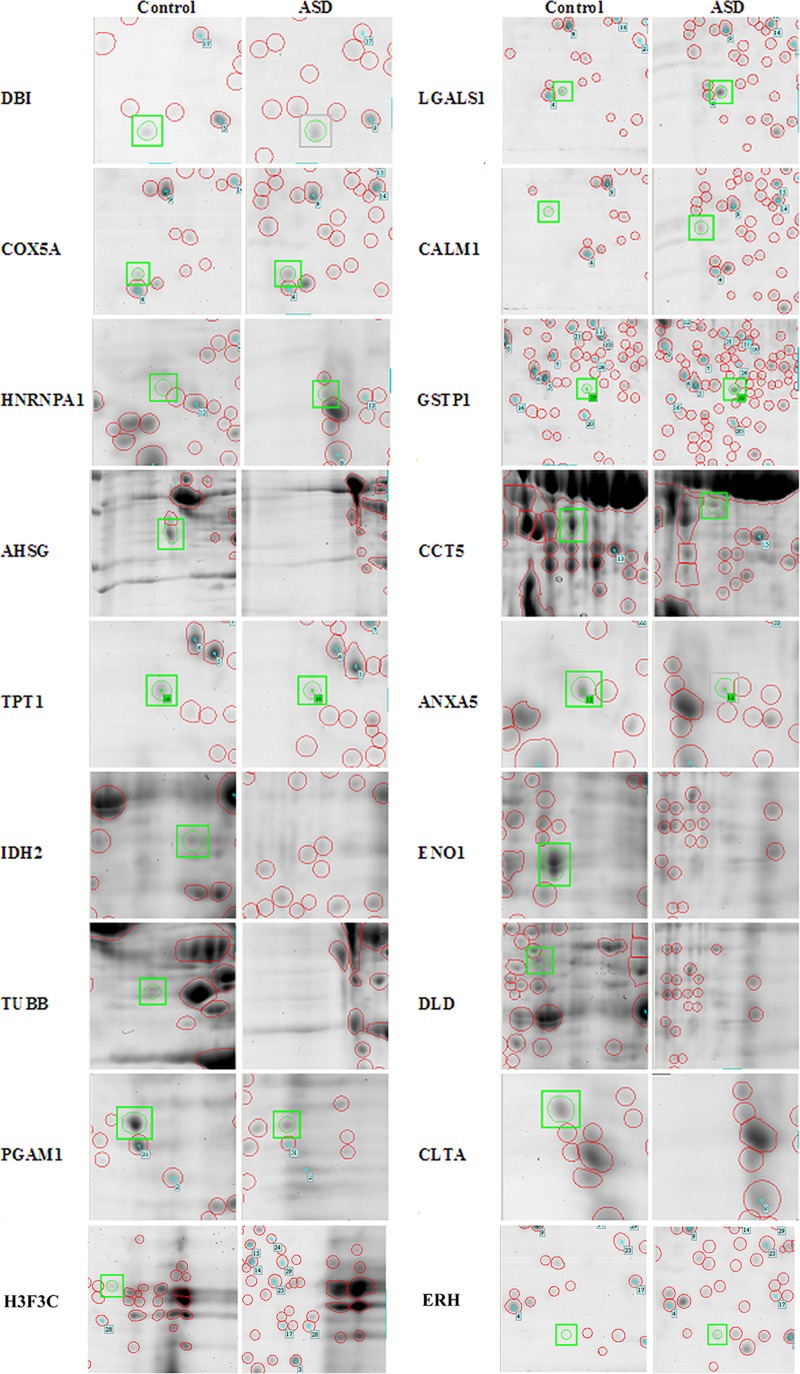
Differentially expressed protein spots in ASD with severe language impairment compared with age-matched controls. Representative images show differentially expressed protein spots identified through 2D gel electrophoresis in the ASD with severe language deficit group compared with sex-/age-matched controls. The red circles show detectable proteins in the gel, and the green circles show significantly differentially expressed protein spots based on statistical analysis via ANOVA (P-value < 0.05).

Because the differentially expressed transcripts in the G3 (red) ASD subgroup (i.e., ASD with severe language impairment) were significantly associated with “autism” based on IPA analysis, we selected this subgroup for further investigation of proteome profiles. Two-dimensional gel electrophoresis analysis of LCLs derived from six individuals with ASD in the G3 (red) subgroup and six sex-/age-matched unaffected controls was performed. A total of 527 protein spots were detected via 2D-gel electrophoresis analysis. Among these protein spots, as many as 82 spots were significantly differentially expressed (ANOVA; P-value < 0.05), as shown in **S4 Table**. A total of 18 protein spots that exhibited a greater than twofold up- or downregulation in the ASD with severe language impairment group when compared to controls were selected for further protein identification through LC-MS/MS analysis (**Fig 6 and S1 Fig)**. To identify the proteins, the MS/MS spectra data corresponding to peptide m/z values from the digestion of each protein spot by trypsin were analyzed using the MASCOT Peptide Mass Fingerprint program in the MASCOT Server (http://www.matrixscience.com/). For each sample, the MASCOT score was calculated to determine the probability that the protein prediction was a random event (**Table 4**).

**Table 4:** List of the top differentially expressed proteins in ASD with severe language impairment.

No.	Predicted Proteins	Description	Fold change (ASD/Control)	P-value (ANOVA; ASD vs Control)	MASCOT Scores of the Predicted Proteins
1	DLD	Dihydrolipoyl dehydrogenase, mitochondrial	↓ 0.097	5.95E-06	666
2	IDH2	Isocitrate dehydrogenase [NADP], mitochondrial	↓ 0.088	1.59E-04	629
3	TPT1	Translationally controlled tumor protein	↓ 0.080	2.03E-04	92
4	ANXA5	Annexin A5	↓ 0.092	6.26E-04	308
5	CCT5	T-complex protein 1 subunit epsilon	↓ 0.259	8.47E-04	545
6	COX5A	Cytochrome c oxidase subunit 5A, mitochondrial	↑ 2.324	1.73E-03	233
7	LGALS1	Galectin-1	↑ 2.354	3.63E-03	612
8	GSTP1	Glutathione S-transferase P	↑ 3.132	3.77E-03	57
9	HNRNPA1	40S ribosomal protein SA	↑ 3.000	4.31E-03	53
10	PGAM1	Phosphoglycerate mutase 1 (brain isoform)	↓ 0.149	5.39E-03	951
11	TUBB	Tubulin beta chain	↓ 0.083	6.30E-03	557
12	ENO1	Alpha-enolase	↓ 0.017	6.81E-03	339
13	H3F3C	Peptidyl-prolyl cis-trans isomerase A	↓ 0.046	8.80E-03	50
14	DBI	Diazepam-binding inhibitor	↑ 2.407	2.21E-02	116
15	AHSG	Alpha-2-HS-glycoprotein	↓ 0.186	3.13E-02	552
16	ERH	Enhancer of rudimentary homolog	↑ 2.950	3.51E-02	32
17	CLTA	Clathrin light chain A	↓ 0.364	3.65E-02	215
18	CALM1	Calmodulin-1	↑ 3.760	4.33E-02	69

P-values were calculated via ANOVA between the ASD with severe language impairment group and the control group. MASCOT scores were calculated with the MASCOT Peptide Mass Fingerprint program. The MASCOT score is provided as the -10log(P), where P is the absolute probability that the observed match is a random event. The higher the MASCOT score is, the higher the confidence level.

**Fig 7:**
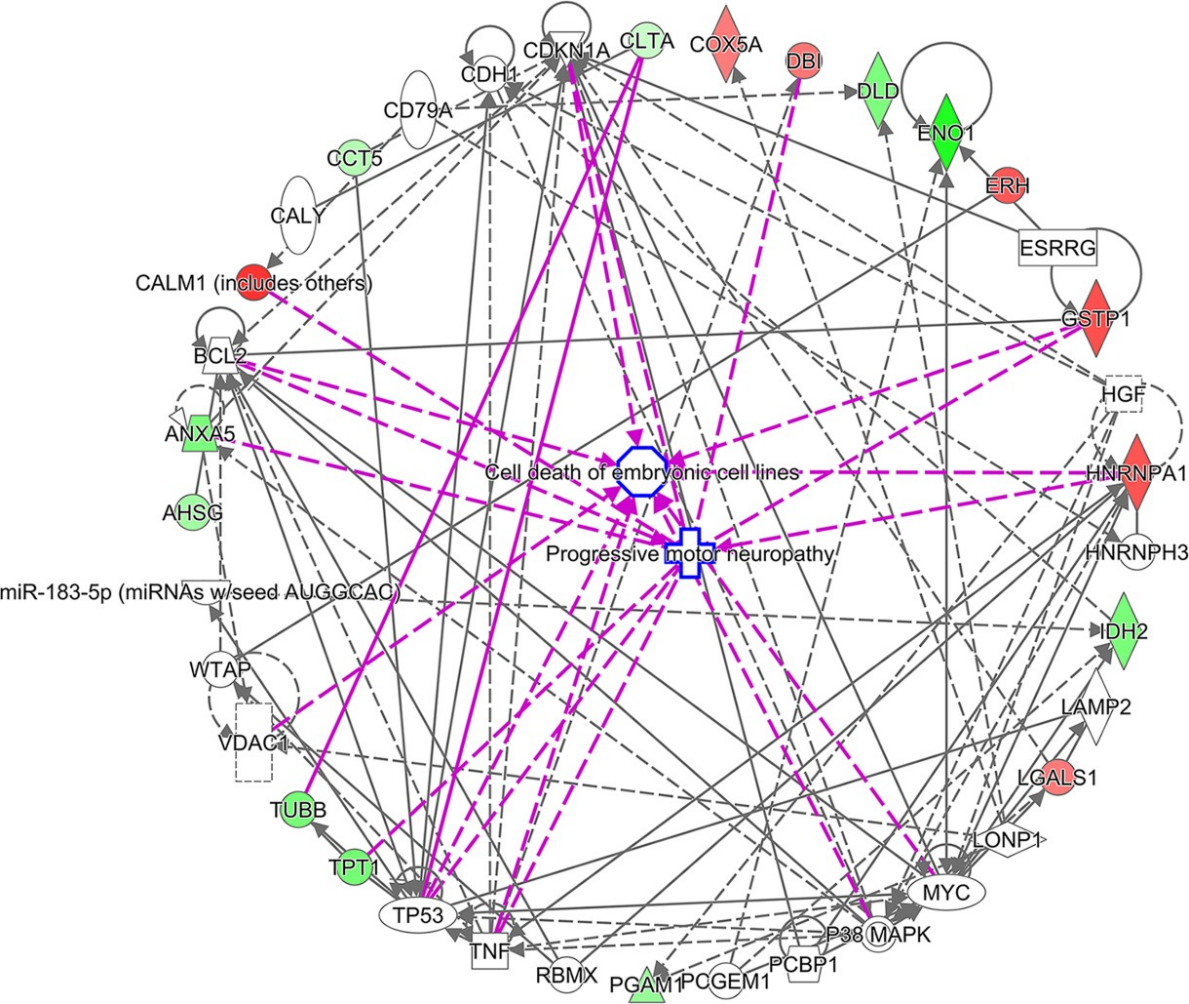
Biological network of the top 18 differentially expressed proteins in the ASD subgroup with severe language impairment identified via 2D-PAGE, followed by LC-MS/MS analysis. We employed the list of predicted differential protein spots from the 2D gel coupled with nano-LC-MS/MS to assemble a protein-protein interaction network using Ingenuity Pathway Analysis (IPA) software.

To predict the biological functions associated with these 18 proteins, ontology analysis was conducted using several tools. Predicted functions and phenotypes associated with these proteins were determined using the GeneCards database. Interestingly, as many as 13 of the 18 differentially expressed proteins are involved in neurological functions/disorders (**Table 5**). In addition, 5 of the 18 proteins (i.e., PGAM1, DLD, IDH2, ENO1, and COX5A) are involved in mitochondrial function and energy production, processes that have been implicated in ASD. Furthermore, we searched for these 18 proteins in the AutismKB ASD database [52]. Of the 18 proteins, as many as 14 have been associated with ASD according to the AutismKB database (**Table 5**). IPA revealed that these proteins are associated with endocrine system disorders, neurological disease, lipid metabolism, inflammatory disease, developmental disorders, and psychological disorders (**Table 6**). Detailed IPA results are shown in **S5 Table**. Biological network analysis showed that these proteins interact with one another (**Fig 7**). Interestingly, several proteins were also found to be encoded by differentially expressed transcripts in ASD transcriptome studies (GSE15402, GSE6575, GSE18123, GSE25507 and GSE42133) (**S6 Table**).

**Table 5:** Biological functions and phenotypes associated with the top differentially expressed proteins in ASD with severe language impairment.

Proteins	Description	GeneCards Functions/Phenotypes	AutismKB Databases
AHSG	Alpha-2-HS-glycoprotein	Present in the cortical plate of the immature cerebral cortex, brain development	/
ANXA5	Annexin A5	Calcium channel activity, inflammation, placental anticoagulation	/
CALM1	Calmodulin-1	Calcium-modulated protein	-
CCT5	T-complex protein 1 subunit epsilon	Hereditary sensory and autonomic neuropathy with spastic paraplegia (HSNSP), Increased circadian period length	/
CLTA	Clathrin light chain A	Component of coated vesicles and synaptic vesicles	/
COX5A	Cytochrome c oxidase subunit 5A, mitochondrial	Mitochondrial respiratory chain	/
DBI	Diazepam-binding inhibitor	Diazepam-binding inhibitor via GABAA receptor binding, behavior/neurological phenotype, mortality/aging	-
DLD	Dihydrolipoyl dehydrogenase, mitochondrial	Global/neurodevelopmental delay, cerebellar ataxia, seizure, etc.	/
ENO1	Alpha-enolase	Activator of plasminogen on the cell surface of several cell types, such as leukocytes and neurons	/
ERH	Enhancer of rudimentary homolog	A component of the methylosome, targeting proteins to the survival of motor neurons (SMN) complex for assembly into small nuclear ribonucleoprotein (snRNP) core particles	-
GSTP1	Glutathione S-transferase P	Detoxification, decreased NANOG and OCT4 protein expression	/
H3F3C	Peptidyl-prolyl cis-trans isomerase A	Chromatin/nucleosome remodeling	/
HNRNPA1	40S ribosomal protein SA	Pre-mRNA processing, early-onset Paget disease, frontotemporal dementia	/
IDH2	Isocitrate dehydrogenase [NADP], mitochondrial	Global/neurodevelopmental delay, seizure, etc.	/
LGALS1	Galectin-1	Expressed in brain endothelial cells, immune tolerance in pregnancy, enhanced apoptosis, autophagy	/
PGAM1	Phosphoglycerate mutase 1 (brain isoform)	Corticobasal degeneration	-
TPT1	Translationally controlled tumor protein	Allergic hypersensitivity disease, paraneoplastic cerebellar degeneration	/
TUBB	Tubulin beta chain	Mutations in this gene cause complex cortical dysplasia, with other brain malformations	/

Biological functions and phenotypes associated with each protein were obtained from the GeneCards Database and AutismKB (/ = present in the database).

**Table 6:** Top diseases associated with differentially expressed proteins in ASD with severe language impairment.

Category	P-value	Proteins
Endocrine System Disorders	5.93E-06–4.16E-02	TUBB, COX5A, ANXA5, GSTP1, AHSG, ENO1, CCT5
Neurological Disease	3.19E-05–3.72E-02	HNRNPA1, DLD, CALM1, TUBB, LGALS1, TPT1, ANXA5, DBI, GSTP1, H3F3C, CCT5, IDH2
Lipid Metabolism	6.25E-05–1.47E-02	LGALS1, ANXA5, DBI, GSTP1
Inflammatory Disease	4.95E-04–4.17E-02	HNRNPA1, LGALS1, ERH, ANXA5, GSTP1, AHSG, ENO1
Developmental Disorder	9.24E-04–1.06E-02	HNRNPA1, DLD, CALM1, TUBB, IDH2
Psychological Disorders	4.61E-03–3.72E-02	HNRNPA1, TUBB, LGALS1, TPT1, DBI, GSTP1

The list of 18 differentially expressed proteins identified via 2D-PAGE and LC-MS/MS analysis was uploaded for Ingenuity Pathway Analysis, and diseases associated with these proteins were predicted. P-values were calculated using Fisher’s exact test.

#### Overlap analysis of proteome-transcriptome profiles

**Fig 8:**
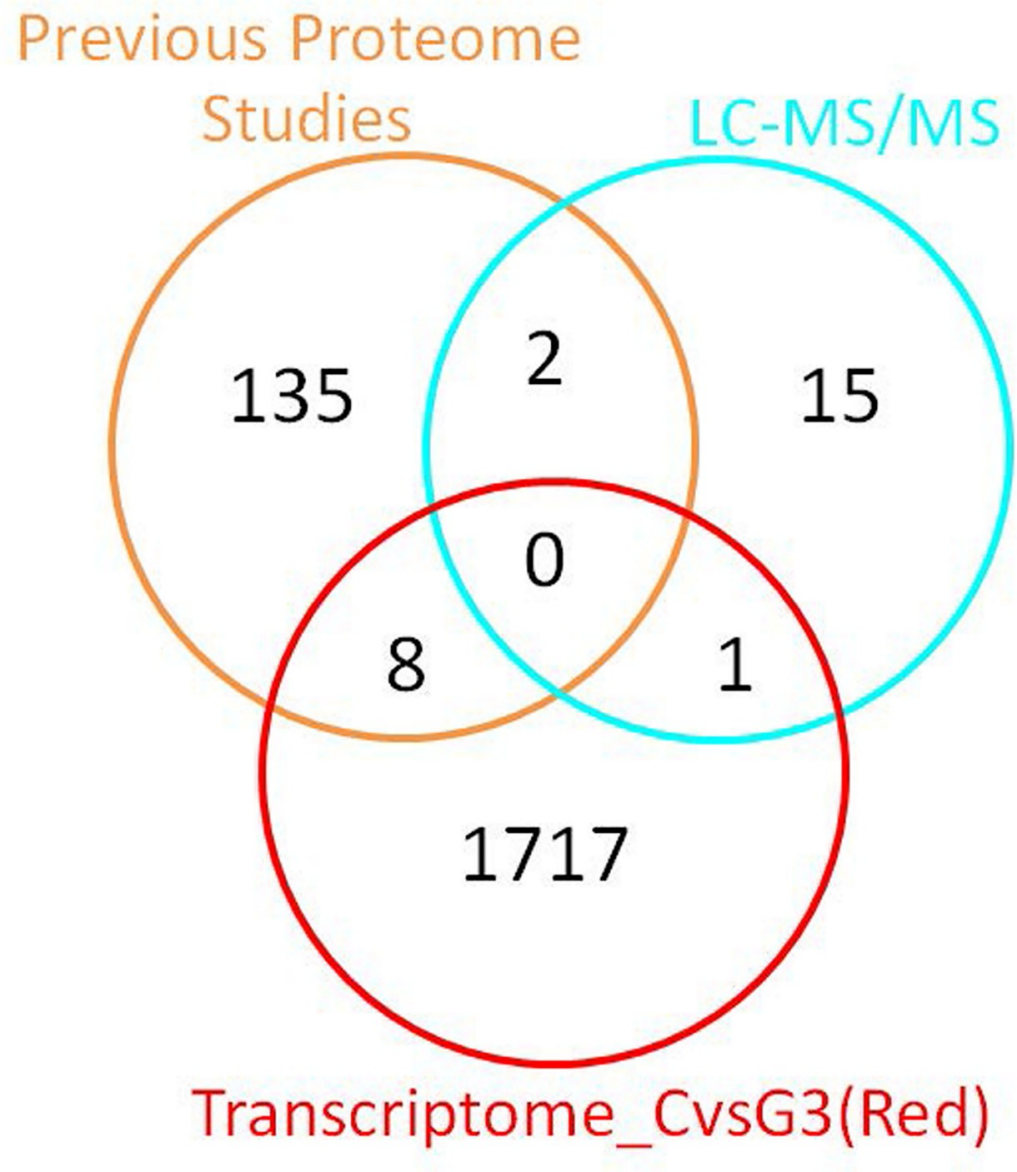
Overlap analysis of the top differentially expressed proteins identified via 2D-PAGE and LC-MS/MS analysis, differentially expressed transcripts, and significant proteins from previous proteomic studies. This Venn diagram shows the number of overlapping transcripts/proteins among the three types of analyses. The list of significant proteins from previous proteomic studies was obtained from the studies listed in **Table 2**.

We next determined whether the differentially expressed proteins identified via our proteome profiling analysis have been identified in previous proteome studies and/or are related to the differentially expressed transcripts identified in the transcriptome analysis. We therefore compared the list of 18 differentially expressed proteins identified via 2D-PAGE followed by LC-MS/MS analysis with the list of proteins from previous proteome studies and the list of differentially expressed transcripts in patients with ASD with severe language impairment (**Fig 8 and Table 7**). Among the 18 top differentially expressed proteins, isocitrate dehydrogenase (IDH2) was also differentially expressed at the transcript level, and two proteins (ENO1, AHSG) have been identified as significantly affected proteins in previous proteome studies.

**Table 7:** Overlap analysis of the top differentially expressed proteins identified via 2D-PAGE and LC-MS/MS analysis, differentially expressed transcripts, and significantly affected proteins from previous proteomic studies.

Comparison	Total	Proteins	LC-MS/MS	Transcriptome_CvsG3(Red)	Previous Proteome [Ref]
LC-MS/MS vs Previous Proteome	2	ENO1	↓ -5.909	NA	↓ -1.743 [31]
		AHSG	↓ -2.428	NA	↓NA [35]
LC-MS/MS vs Transcriptome CvsG3(Red)	1	IDH2	↓ -3.493	↓ -0.294	NA
Previous Proteome vs Transcriptome CvsG3(Red)	8	FN1	NA	↑ 0.478	↑ 2.363 [31]
		C5	NA	↑ 0.313	↑ 2.127 [31]
		VTN	NA	↑ 0.181	↑ 2.390 [31]
		IGFBP5	NA	↑ 0.363	↑ 0.024 [27]
		PPP1R2	NA	↓ -0.316	↓ NA [32]
		PTGDS	NA	↓ -0.302	↑ NA [35]
		MBP	NA	↓ -0.290	↑ 0.495 [25]
		APOA1	NA	↑ 0.285	↑ 0.024 [28]

This table shows the number of overlapping transcripts/proteins among the three types of analyses and the log_2_ expression ratios (ASD/control). The list of significant proteins from previous proteomic studies was obtained from the studies listed in **Table 2**.

#### Western blot analysis

**Fig 9:**
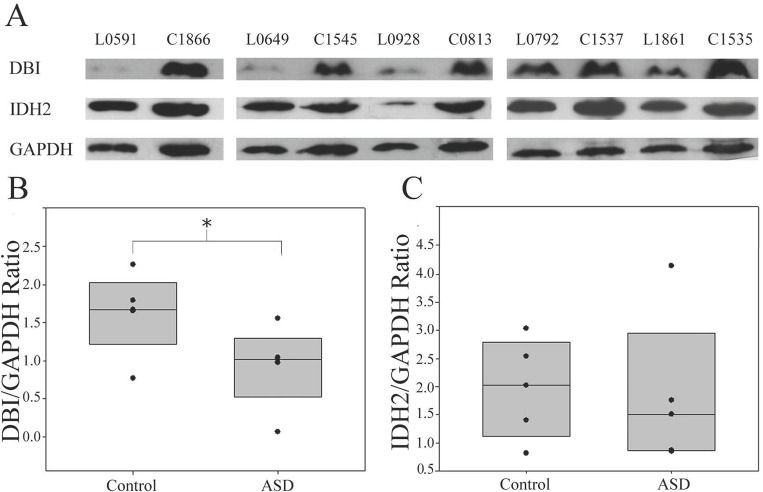
DBI protein levels are decreased in ASD with severe language impairment. Western blot analysis of LCLs from individuals with ASD with severe language impairment and sex-/age-matched controls was conducted using GAPDH as the endogenous control. P-values were determined with the two-tailed t-test. The error bars represent the S.E.M. The protein expression of DBI was significantly reduced in LCLs from individuals with ASD with severe language impairment (P-value < 0.05).

To validate the dysregulation of the proteins identified via 2D gel electrophoresis, DBI and IDH2 proteins were selected for the Western blot analysis (**Fig 9**). Changes in DBI have been reported in ASD urine samples [35], and loss of function of this gene/protein causes disruption of social interaction [57], a key behavioral domain that is negatively impacted in ASD. IDH2 is disrupted at the transcript level and is involved in energy production in association with ASD [16]. The results of the Western blot analysis showed that the DBI protein level was significantly decreased in individuals with ASD with severe language impairment compared to sex-/age-matched controls (P-value < 0.05) but was not significantly changed in the other ASD subgroups (**S2 Fig**). The expression level of IDH2 in individuals with ASD with severe language impairment tended to be lower than that in the age-matched controls but was not changed in the other subgroups (**S2 Fig**). Although the reduction of IDH2 in ASD was not statistically significant, it was notable that IDH2 expression in four individuals with ASD with severe language impairment was lower than the average IDH2 level in the control group.

To determine whether a correlation exists between the protein levels of DBI and IDH2 and ASD-related clinical phenotypes, we conducted a correlation analysis between the protein levels of DBI and IDH2 and 123 ADI-R scores of individuals with ASD from all four subgroups (**Table 8**). Although significant differences in the ADI-R items were not observed after multiple testing correction using the Benjamini-Hochberg procedure (FDR = 0.05), it is interesting to note that the DBI protein levels were correlated with certain ADI-R items (nominal P-value < 0.05), including direct gaze, visuospatial ability, memory skill, musical ability, drawing skill, reading ability, and computational ability. Moreover, the IDH2 protein level were correlated with the overall level of language, reciprocal conversation, neologisms/idiosyncratic language, verbal rituals, head shaking, initiation of appropriate activities, undue general sensitivity to noise, other complex mannerisms or stereotyped body movements, gait, aggression to non-caregivers or nonfamily members, visuospatial ability, memory skill, reading ability, and computational ability.

**Table 8:** Correlation analysis between the levels of DBI or IDH2 proteins and ADI-R scores.

**DBI VS ADI-R Scores**				
**ADI-R Items**	**ADI-R Description**	**Pearson Correlation**	**P-value**	**Benjamini-Hochberg P-value**
GAZE5	Direct gaze	0.443	0.014	0.190
CVISSPZ	Visuospatial ability (i.e., in puzzles, jigsaws, shapes, patterns, etc.)	-0.520	0.003	0.190
EVISSPZ	Visuospatial ability (i.e., in puzzles, jigsaws, shapes, patterns, etc.)	-0.507	0.004	0.190
CMEMZ	Memory skill (accurate memory for detail, as of dates or timetables)	-0.462	0.010	0.190
EMEMZ	Memory skill (accurate memory for detail, as of dates or timetables)	-0.450	0.011	0.190
CMUSICZ	Musical ability (recognition, composition, absolute pitch or performance)	-0.424	0.020	0.222
EMUSICZ	Musical ability (recognition, composition, absolute pitch or performance)	-0.411	0.022	0.224
CDRAWZ	Drawing skill (unusually skilled use of perspective or creative approach)	-0.453	0.012	0.190
EDRAWZ	Drawing skill (unusually skilled use of perspective or creative approach)	-0.437	0.014	0.190
CREADZ	Reading ability (e.g., early sight reading)	-0.436	0.016	0.195
EREADZ	Reading ability (e.g., early sight reading)	-0.401	0.025	0.235
CCOMPUZ	Computational ability (e.g., mental arithmetic)	-0.463	0.010	0.190
ECOMPUZ	Computational ability (e.g., mental arithmetic)	-0.440	0.013	0.190
**IDH2 VS ADI-R Scores**				
**ADI-R Items**	**ADI-R Description**	**Pearson Correlation**	**P-value**	**Benjamini-Hochberg P-value**
LEVELL	Overall level of language	0.385	0.030	0.312
CCONVER	Reciprocal conversation (at whatever verbal level of complexity possible)	0.395	0.025	0.312
CNEOID	Neologisms/idiosyncratic language	0.410	0.022	0.312
ENEOID	Neologisms/idiosyncratic language	0.451	0.011	0.312
EVERRIT	Verbal rituals	0.387	0.029	0.312
HSHAKE5	Head shaking	0.368	0.046	0.312
INITIA5	Initiation of appropriate activities	0.513	0.005	0.312
CNOISE	Undue general sensitivity to noise	0.456	0.011	0.312
COTHMAN	Other complex mannerisms or stereotyped body movements (do not include isolated rocking)	0.380	0.035	0.312
EOTHMAN	Other complex mannerisms or stereotyped body movements (do not include isolated rocking)	0.372	0.039	0.312
CGAIT	Gait	0.451	0.012	0.312
CAGGOTH	Aggression to noncaregivers or nonfamily members	0.483	0.031	0.312
CVISSPZ	Visuospatial ability (i.e., in puzzles, jigsaws, shapes, patterns, etc.)	-0.368	0.045	0.312
EVISSPZ	Visuospatial ability (i.e., in puzzles, jigsaws, shapes, patterns, etc.)	-0.386	0.032	0.312
CMEMZ	Memory skill (accurate memory for detail, as of dates or timetables)	-0.406	0.026	0.312
EMEMZ	Memory skill (accurate memory for detail, as of dates or timetables)	-0.423	0.018	0.312
EREADZ	Reading ability (e.g., early sight reading)	-0.373	0.039	0.312
ECOMPUZ	Computational ability (e.g., mental arithmetic)	-0.369	0.041	0.312

Pearson correlation analysis was conducted using the levels of DBI/GAPDH or IDH2/GAPDH and the scores of 123 ADI-R items of individuals with ASD from all four subgroups. Only ADI-R items that showed a significant correlation (nominal P-value < 0.05) are shown. Multiple testing corrections was also conducted using the Benjamini-Hochberg procedure (FDR = 0.05).

## Discussion

In the present study, we integrated clinical phenotype subgrouping through a cluster analysis with transcriptome-proteome profiling analyses. Here, 2D-SDS PAGE followed by LC-MS/MS analysis were used to identify proteins that were differentially expressed in a subgroup of individuals with ASD with severe language impairment. Because the expression of two of these proteins was significantly correlated with a number of specific behavioral manifestations of ASD, these proteins as well as other differentially expressed proteins might serve as candidate proteins for future research into the molecular mechanisms of ASD or the discovery of ASD biomarkers.

### Proteomic analysis reveals novel differentially expressed proteins in a subtype of ASD

PCA and HCL analyses of ADI-R scores showed that the 85 ASD cases could be divided into four subgroups/clusters based on their clinical phenotypes **(Fig 2)**. Reanalysis of the transcriptome profile data of LCLs derived from ASD cases revealed specific transcriptome profiles that were disrupted in each subgroup **(Fig 3 and S2 Table)**. Notably, the protein products of certain differentially expressed transcripts identified in LCLs or in blood have been reported to be disrupted in previous proteome analyses of ASD **(Fig 5)**, which prompted us to examine the proteomic profile of LCLs for which transcriptomic data had been already obtained [16]. We focused on the G3 (red) subgroup because it was the only subgroup with differentially expressed transcripts that were significantly associated with the term “autism” by the IPA. A proteome profiling analysis of LCLs from this subgroup of ASD patients and sex-/age-matched controls using 2D-PAGE showed that as many as 82 protein spots were differentially expressed in this subgroup **(S4 Table)**. Based on at least a 2-fold change in spot intensity **(Fig 6)**, a total of 18 spots were selected for protein identification via LC-MS/MS analysis. **Table 4** shows the list of proteins identified by the LC-MS/MS analyses with high confidence levels, as indicated by the high MASCOT scores. Interestingly, 14 of the 18 identified proteins have been associated with neurological functions/diseases **(Table 4, Table 5, Fig 7, and S5 Table)**. A Western blot analysis of the LCLs further showed that the DBI and IDH2 proteins were specific for the severely language-impaired group, suggesting their potential as protein biomarkers for this subgroup **(Fig 9)**. Interestingly, the expression levels of both proteins showed significant inverse correlations with all six of the savant skill items (visuospatial ability, memory skill, musical ability, drawing skill, reading ability, and computational ability) **(Table 8)**, suggesting their possible involvement in critical neural pathways related to the development of these skills. In addition, the level of IDH2 protein is directly correlated with a number of language items on the ADI-R scoresheet (including overall level of language), complex mannerisms or stereotyped body movements, unusual gait, and auditory sensitivity, which are frequently associated with ASD. Although the association of these genes with these specific ADI-R items is intriguing, further investigations are needed to determine the molecular and/or physiological mechanisms through which deficits in these proteins may increase the manifestation of savant skills and other stereotyped behaviors characteristic of ASD.

IDH2 was also found to be dysregulated at the transcript level, and the proteins AHSG and ENO1 have been reported in previous proteome studies of ASD [31,35]. These findings suggest that certain differentially expressed proteins in LCLs from the ASD subgroup with severe language impairment are reproducible in different ASD cohorts, thus emphasizing the importance of reducing the heterogeneity of ASD by clinical phenotype clustering or other methods in future proteomic research on ASD.

### Comparison of ASD subgroups obtained by cluster analyses of ADI-R scores

Recent transcriptome studies have demonstrated that subgrouping individuals with ASD based on ADI-R scores helps reduce the heterogeneity of ASD populations, thus permitting the identification of differentially expressed genes and molecular mechanisms specific to ASD subpopulations. Subgroup-specific genes might be useful in the development of ASD subtype biomarkers or personalized treatments for specific groups of individuals with ASD [41]. In this study, we used the same subgrouping strategy, by performing cluster analysis of the ADI-R scores of 85 individuals with ASD whose LCLs were previously subjected to a transcriptome profiling analysis using cDNA microarrays [16]. Similar to a previous study by Hu et al. (2009), cluster analyses of the ADI-R scores showed that the individuals with ASD clustered into distinct subpopulations. Our study revealed at least four distinct subgroups, designated G1-4, among the original 85 cases used for the transcriptomic analyses **(Fig 2)**. The HCL analysis revealed that each subgroup exhibited distinct patterns of ADI-R scores, with the G3 (red) subgroup consisting of individuals who represented a subset of the original ASD subgroup identified as “severely language-impaired”[16]. A closer examination of the HCL data in **Fig 2A** reveals that the G2 (green) subgroup represents individuals with severe language impairment who also have notable savant skills (notably high ADI-R scores in the 12 columns at the extreme right of the graph for the G2 cluster), and they are separated from the language-impaired G3 group without savant skills. The remaining two subgroups, G1 (blue) and G4 (yellow), show some discrepancies with the blue and yellow subgroups from the earlier transcriptomic study. The source of the minor discrepancies can be explained in part by the number of cases included in the cluster analyses. For the original transcriptomic study [16], cluster analyses were performed on ADI-R scores from 1,954 individuals with ASD which resulted in the current G2 and G3 subgroups being represented within one severely language-impaired subgroup [41]. Three other subgroups were identified, with two of them similar in phenotype to the G1 and G4 subgroups identified in this study. The remaining subgroup (called “green” or “intermediate” in the original paper) was not included in the expression profiling analyses and thus is not represented here. The present study shows that it is possible to further divide the ASD population into more clinically defined subgroups by applying hierarchical cluster analyses of ADI-R scores for smaller groups of individuals with ASD (85 cases vs. 1,954 cases) or by selecting smaller clusters from the dendrogram resulting from HCL analyses of larger samples.

### Genes and pathways implicated by functional and network prediction analyses

DBI has not been identified in any other blood-based proteomic study of ASD, but a recent study indicated that DBI protein was differentially expressed among as many as 231 proteins in urine from individuals with ASD [35]. DBI is also known as acyl-CoA-binding protein (ACBP) or endozepine (EP). DBI can displace diazepam from the benzodiazepine (BZD) recognition site located in the GABA type A receptor and act as a neuropeptide to modulate its action [58]. DBI is highly expressed in several regions of the brain, including the cerebellar cortex, area postrema, and ependyma of the third ventricle [59]. A recent study in mice reported that genetic loss of DBI leads to reduced social interest in both males and females, with a greater effect in males than in females [57]. Moreover, male and female mice lacking DBI exhibit increased repetitive grooming [57]. These altered behaviors are key phenotypes of ASD, and the sex differences in the effects of DBI loss on social behavior may be related to the sex bias of ASD, which deserves further investigation. Moreover, in addition to LCLs, the reduction of DBI protein levels should be further confirmed in peripheral blood or brain samples from individuals with ASD with severe language impairment in a larger cohort.

The other differentially expressed proteins in LCLs from individuals with ASD with severe language impairment that were reproducible were AHSG, ENO1, and CALM1. AHSG, or fetuin-A, is found in the peripheral blood and is produced by the liver [60]. The protein is involved in several processes, including brain development, endocytosis, and the formation of bone tissue [61]. A proteomic study using liquid chromatography-electrospray ionization-mass spectrometry (LC-ESI-MS) analysis revealed the upregulation of AHSG in the serum of low-functioning ASD individuals compared with controls [26]. Moreover, a genomic linkage scan performed in a study of SNPs in individuals with ASD also identified *AHSG* as a potential candidate gene for a chromosome 3q26.31–q27.3 region mutation [62]. ENO1 and CALM1 were reported to be decreased in another previous proteome study using isobaric tags for relative and absolute quantitation (iTRAQ)-based mass spectrometry analysis of plasma from patients with ASD and age-matched unaffected individuals [31]. ENO1 (alpha-enolase) is a multifunctional enzyme that plays a role in glycolysis and functions as an activator of plasminogen on the cell surface of leukocytes and neurons. CALM1 (calmodulin 1) is a calcium-binding protein involved in the regulation of a number of ion channels and enzymes through calcium binding and is important for neurological functions. Like DBI, these proteins deserve further study.

In addition to these differentially expressed proteins identified in previous proteome studies, other proteins have also been linked to ASD. One example is TUBB, which is the major constituent of microtubules and is highly expressed in the developing brain. Mutations in the gene encoding this protein lead to cognitive impairment with motor and language delay, ataxia, and severely delayed psychomotor development, all of which are associated with ASD [63,64]. Notably, a reduction in TUBB protein levels was also observed in the ASD G3 subgroup, whose members exhibited severe language impairment.

In addition to neurological functions, several differentially expressed proteins are involved in mitochondrial functions and energy production, which have been reported to be negatively impacted in ASD [see reviews in [65,66]]. For example, IDH2 (isocitrate dehydrogenase) is found in the mitochondria and plays a role in intermediary metabolism and energy production by catalyzing the oxidative decarboxylation of isocitrate to 2-oxoglutarate. In the present study, we found that IDH2 transcript and protein levels were dysregulated in ASD with severe language impairment. The expression of mutated IDH2 has been associated with white matter abnormalities throughout the central nervous system (CNS) and with muscular dystrophy in transgenic adult mice [67]. Both of these conditions have been associated with ASD [68–70]. Western blot analysis of protein from the ASD with severe language impairment group showed that IDH2 protein levels tended to be lower in the ASD subgroup, although this difference was not statistically significant **(Fig 9)**. The potential role of this protein should be further examined in a larger cohort.

Other proteins related to mitochondrial functions identified by this study included PGAM1, DLD, ENO1, and COX5A. PGAM1 is an enzyme of the glycolytic pathway that catalyzes the conversion of 3-phosphoglycerate to 2-phosphoglycerate. DLD functions as the E3 subunit of three mitochondrial multienzyme complexes: pyruvate dehydrogenase (PDH), alpha-ketoglutarate dehydrogenase (α-KGDH) and branched chain 2-oxoacid dehydrogenase (BCKDH). COX5A is the nuclear-encoded Va subunit of the human mitochondrial respiratory chain enzyme. Abnormalities in these important mitochondrial proteins might be responsible for susceptibility to ASD and therefore require further investigation.

### Limitations and future directions

Our study shows that ASD subgrouping performed to reduce clinical heterogeneity can identify proteins with the potential for use as molecular marker candidates in future studies. All LCLs used in this study were obtained from the AGRE. The control LCLs were derived from male unaffected individuals who do not have ASD. These LCLs have been used for gene expression profiling analysis using high-throughput microarrays in a previous study [16], allowing us to obtain transcriptome data for comparison with the proteome data in this study and others. A limitation of this study with regard to proteomic data is that we combined the lists of differentially expressed proteins from different proteomics studies on ASD for comparison with differentially expressed genes from a handful of transcriptomics analyses. However, a risk of bias and lack of consistency may occur because of the different analytical techniques, types of tissues, and other confounding factors among the various studies. In future work, a comprehensive meta-analysis and/or a systematic review of published proteome profiles might be conducted to generate a list of differentially expressed proteins in each type of tissue. Another limitation may be the unknown ethnicity of the majority of the individuals whose LCLs were used in this study. Although we do not have information about the specific ethnicity of the individuals in this study, most individuals were Caucasian and described as “Not Hispanic or Latino” according to the AGRE. Whether ancestry has a notable impact on proteome and/or transcriptome profiles has not been clarified; therefore, the findings from this study should be confirmed in a larger cohort with age-, sex-, and ethnicity-matched controls in the future.

## Conclusions

The findings of this study demonstrate that subgrouping individuals with ASD based on cluster analyses of ADI-R scores is useful for identifying protein candidates for ASD, particularly in the phenotypic subgroup of individuals with ASD with severe language impairment. Integrated transcriptome-proteome profiling analyses of LCLs revealed that differentially expressed transcripts in LCLs from individuals with ASD with severe language impairment were significantly associated with proteins reported to be affected in patients with ASD based on proteomics analyses. Thus, several of the altered proteins identified in LCLs might reflect molecular changes in patients, and LCLs may be employed as a surrogate tissue for screening candidate proteins in ASD. As the majority of differentially expressed genes may not correlate with differential protein levels, additional proteomic studies are clearly needed to identify protein markers and the molecular mechanisms underlying ASD. Proteome profiling of LCLs from individuals with ASD with severe language impairment revealed several dysregulated proteins. These proteins included DBI, which was significantly decreased in ASD cases compared with sex-/age-matched controls and which may serve as a candidate protein for future studies of molecular mechanisms or biomarker discovery in ASD.

## Supporting information

S1 Table Demographic information of individuals with ASD and unaffected individuals included in this study. (XLS)

S2 Table List of significantly differentially expressed transcripts in the ASD group and in each subgroup compared to the unaffected control group. (XLS)

S3 Table Significantly differentially expressed genes in each subgroup that were associated with the IPA categories “developmental disorder” and “neurological disease”. (XLS)

S4 Table Differentially expressed spots from the 2D-PAGE and LC-MS/MS analyses. (XLS)

S5 Table Top diseases/disorders associated with 18 differentially expressed proteins in ASD with severe language impairment. (XLS)

S6 Table Overlapping proteins between the 18 differentially expressed proteins from the LC-MS/MS analysis and those proteins encoded by transcripts found to be differentially expressed in previous ASD transcriptome studies. (XLS)

S1 Fig Representative images of the 2D-PAGE analysis. (PDF)

S2 Fig Western blot analysis of the DBI and IDH2 proteins in the ASD subgroups. (PDF)

## Abbreviations

2D-PAGE: Two-Dimensional Polyacrylamide Gel Electrophoresis
ACBP: Acyl-CoA-Binding Protein
aCGH: Array Comparative Genomic Hybridization
ACN: Acetonitrile
ADI-R: Autism Diagnostic Interview, Revised
AGRE: Autism Genetic Resource Exchange
AHSG: Alpha-2-HS-glycoprotein
ANXA5: Annexin A5
ASD: Autism Spectrum Disorder
ApoA1: Apolipoprotein A1
ApoA4: Apolipoprotein A4
ATP: Adenosine Triphosphate
BCKDH: Branched Chain 2-Oxoacid Dehydrogenase
BSA: Bovine Serum Albumin
BZD: Benzodiazepine
CALM1: Calmodulin-1
CCT5: T-Complex Protein 1 Subunit Epsilon
CEP290: Centrosomal Protein 290
CDC: Centers for Disease Control and Prevention
CHAPS: 3-[(3-Cholamidopropyl)Dimethylammonio]-1-Propanesulfonate
CLTA: Clathrin light chain A
CNS: Central Nervous System
CNV: Copy Number Variation
COX5A: Cytochrome c oxidase subunit 5A
DBI: Diazepam-Binding Inhibitor
DEGs: Differentially Expressed Genes
DLD: Dihydrolipoyl Dehydrogenase
DSM-5: The Diagnostic and Statistical Manual of Mental Disorders, Fifth Edition
DTT: Dithiothreitol
ECL: Enhanced Chemiluminescence
EIF4G1: Eukaryotic Translation Initiation Factor 4 Gamma 1
ENO1: Alpha-Enolase
EP: Endozepine
ERH: Enhancer of Rudimentary Homolog
FISH: Fluorescence In Situ Hybridization
GABA: Gamma-Aminobutyric Acid
GAPDH: Glyceraldehyde-3-Phosphate Dehydrogenase
GSTP1: Glutathione S-transferase P
GST: Glutathione S-transferase
H3F3C: Peptidyl-Prolyl Cis-Trans Isomerase A
HCL: Hierarchical Clustering
HNRNPA1: 40S Ribosomal Protein SA
HRP: Horseradish Peroxidase
IAA: Iodoacetamide
IDH2: Isocitrate Dehydrogenase
IKKα: I Kappa K alpha
α-KGDH: Alpha-Ketoglutarate Dehydrogenase
IPA: Ingenuity Pathway Analysis
iTRAQ: Isobaric Tag for Relative and Absolute Quantitation
LCLs: Lymphoblastoid Cell Lines
LC-MS/MS: Liquid Chromatography Mass Spectrometer
LC-ESI-MS: Liquid Chromatography-Electrospray Ionization-Mass Spectrometry
LGALS1: Galectin-1
MeV: Multiple Experiment Viewer
miRNA: MicroRNAs
MRI: Magnetic Resonance Imaging
NCBI: National Center for Biotechnology Information
PCA: Principal Component Analysis
PDH: Pyruvate Dehydrogenase
PGAM1: Phosphoglycerate Mutase 1
PON1: Paraoxanase/Arylesterase 1
PRKCI: Protein Kinase C Iota
PTPN11: Tyrosine-Protein Phosphatase Non-Receptor Type 11
PVDF: Polyvinylidene Fluoride
RPMI: Roswell Park Memorial Institute
SDS-PAGE: Sodium Dodecyl Sulfate Polyacrylamide Gel Electrophoresis
SNP: Single Nucleotide Polymorphism
TBST: Tris-Buffered Saline
TCA: Tricarboxylic Acid Cycle
TPT1: Translationally Controlled Tumor Protein
TUBB: Tubulin Beta Chain
Tyk2: Tyrosine Kinase 2

## References

[1] Vorstman JA, Parr JR, Moreno-De-Luca D, Anney RJ, Nurnberger JI Jr, et al (2017) Autism genetics: opportunities and challenges for clinical translation. Nature Reviews Genetics 18: 362 10.1038/nrg.2017.4 28260791

[2] Bölte S, Girdler S, Marschik PB (2018) The contribution of environmental exposure to the etiology of autism spectrum disorder. Cellular and Molecular Life Sciences: 1–23. 10.1007/s00018-018-2988-4 PMC6420889 30570672

[3] Moosa A, Shu H, Sarachana T, Hu VW (2018) Are endocrine disrupting compounds environmental risk factors for autism spectrum disorder? Hormones and behavior 101: 13–21. 10.1016/j.yhbeh.2017.10.003 29042182 PMC5913002

[4] Thongkorn S, Kanlayaprasit S, Jindatip D, Tencomnao T, Hu VW, et al (2019) Sex Differences in the Effects of Prenatal Bisphenol A Exposure on Genes Associated with Autism Spectrum Disorder in the Hippocampus. Scientific reports 9: 3038 10.1038/s41598-019-39386-w 30816183 PMC6395584

[5] Baio J, Wiggins L, Christensen DL, Maenner MJ, Daniels J, et al (2018) Prevalence of autism spectrum disorder among children aged 8 years—autism and developmental disabilities monitoring network, 11 sites, United States, 2014. MMWR Surveillance Summaries 67: 1. 10.15585/mmwr.ss6706a1 PMC5919599 29701730

[6] American Psychiatric Association (2013) Diagnostic and statistical manual of mental disorders (DSM-5®): American Psychiatric Pub.

[7] DiCicco-Bloom E, Lord C, Zwaigenbaum L, Courchesne E, Dager SR, et al (2006) The developmental neurobiology of autism spectrum disorder. J Neurosci 26: 6897–6906. 10.1523/JNEUROSCI.1712-06.2006 16807320 PMC6673916

[8] Tangsuwansri C, Saeliw T, Thongkorn S, Chonchaiya W, Suphapeetiporn K, et al (2018) Investigation of epigenetic regulatory networks associated with autism spectrum disorder (ASD) by integrated global LINE-1 methylation and gene expression profiling analyses. PLoS One 13: e0201071 10.1371/journal.pone.0201071 30036398 PMC6056057

[9] Saeliw T, Tangsuwansri C, Thongkorn S, Chonchaiya W, Suphapeetiporn K, et al (2018) Integrated genome-wide Alu methylation and transcriptome profiling analyses reveal novel epigenetic regulatory networks associated with autism spectrum disorder. Mol Autism 9: 27 10.1186/s13229-018-0213-9 29686828 PMC5902935

[10] Talebizadeh Z, Arking DE, Hu VW (2013) A Novel Stratification Method in Linkage Studies to Address Inter- and Intra-Family Heterogeneity in Autism. PLoS One 8: e67569 10.1371/journal.pone.0067569 23840741 PMC3694043

[11] Hu VW (2013) The expanding genomic landscape of autism: discovering the 'forest' beyond the 'trees'. Future Neurol 8: 29–42. 10.2217/fnl.12.83 23637569 PMC3637978

[12] Hu VW (2011) A systems approach towards an understanding, diagnosis and personalized treatment of autism spectrum disorders. Pharmacogenomics 12: 1235–1238. 10.2217/pgs.11.94 21919600

[13] Sarachana T, Xu M, Wu R-C, Hu VW (2011) Sex Hormones in Autism: Androgens and Estrogens Differentially and Reciprocally Regulate RORA, a Novel Candidate Gene for Autism. PLoS ONE 6: e17116 10.1371/journal.pone.0017116 21359227 PMC3040206

[14] Nguyen A, Rauch TA, Pfeifer GP, Hu VW (2010) Global methylation profiling of lymphoblastoid cell lines reveals epigenetic contributions to autism spectrum disorders and a novel autism candidate gene, RORA, whose protein product is reduced in autistic brain. Faseb j 24: 3036–3051. 10.1096/fj.10-154484 20375269 PMC2909294

[15] Hu VW, Frank BC, Heine S, Lee NH, Quackenbush J (2006) Gene expression profiling of lymphoblastoid cell lines from monozygotic twins discordant in severity of autism reveals differential regulation of neurologically relevant genes. BMC Genomics 7: 118 10.1186/1471-2164-7-118 16709250 PMC1525191

[16] Hu VW, Sarachana T, Kim KS, Nguyen A, Kulkarni S, et al (2009) Gene expression profiling differentiates autism case-controls and phenotypic variants of autism spectrum disorders: evidence for circadian rhythm dysfunction in severe autism. Autism Res 2: 78–97. 10.1002/aur.73 19418574 PMC2737477

[17] Hu VW, Lai Y (2013) Developing a Predictive Gene Classifier for Autism Spectrum Disorders Based upon Differential Gene Expression Profiles of Phenotypic Subgroups. N Am J Med Sci (Boston) 6. 10.7156/najms.2013.0603107 PMC3867975 24363828

[18] Hu VW, Nguyen A, Kim KS, Steinberg ME, Sarachana T, et al (2009) Gene expression profiling of lymphoblasts from autistic and nonaffected sib pairs: altered pathways in neuronal development and steroid biosynthesis. PLoS One 4: e5775 10.1371/journal.pone.0005775 19492049 PMC2685981

[19] Sarachana T, Zhou R, Chen G, Manji HK, Hu VW (2010) Investigation of post-transcriptional gene regulatory networks associated with autism spectrum disorders by microRNA expression profiling of lymphoblastoid cell lines. Genome Med 2: 23 10.1186/gm144 20374639 PMC2873801

[20] Talebizadeh Z, Butler MG, Theodoro MF (2008) Feasibility and relevance of examining lymphoblastoid cell lines to study role of microRNAs in autism. Autism Res 1: 240–250. 10.1002/aur.33 19360674 PMC2768334

[21] Abu-Elneel K, Liu T, Gazzaniga FS, Nishimura Y, Wall DP, et al (2008) Heterogeneous dysregulation of microRNAs across the autism spectrum. Neurogenetics 9: 153–161. 10.1007/s10048-008-0133-5 18563458

[22] Sarachana T, Hu VW (2013) Genome-wide identification of transcriptional targets of RORA reveals direct regulation of multiple genes associated with autism spectrum disorder. Mol Autism 4: 14 10.1186/2040-2392-4-14 23697635 PMC3665583

[23] Sarachana T, Hu VW (2013) Differential recruitment of coregulators to the RORA promoter adds another layer of complexity to gene (dys) regulation by sex hormones in autism. Mol Autism 4: 39 10.1186/2040-2392-4-39 24119295 PMC4016566

[24] Hu VW, Sarachana T, Sherrard RM, Kocher KM (2015) Investigation of sex differences in the expression of RORA and its transcriptional targets in the brain as a potential contributor to the sex bias in autism. Molecular Autism 6: 7 10.1186/2040-2392-6-7 26056561 PMC4459681

[25] Broek JA, Guest PC, Rahmoune H, Bahn S (2014) Proteomic analysis of post mortem brain tissue from autism patients: evidence for opposite changes in prefrontal cortex and cerebellum in synaptic connectivity-related proteins. Mol Autism 5: 41 10.1186/2040-2392-5-41 25126406 PMC4131484

[26] Corbett BA, Kantor AB, Schulman H, Walker WL, Lit L, et al (2007) A proteomic study of serum from children with autism showing differential expression of apolipoproteins and complement proteins. Mol Psychiatry 12: 292–306. 10.1038/sj.mp.4001943 17189958

[27] Ramsey JM, Guest PC, Broek JA, Glennon JC, Rommelse N, et al (2013) Identification of an age-dependent biomarker signature in children and adolescents with autism spectrum disorders. Mol Autism 4: 27 10.1186/2040-2392-4-27 23915542 PMC3751071

[28] Ngounou Wetie AG, Wormwood K, Thome J, Dudley E, Taurines R, et al (2014) A pilot proteomic study of protein markers in autism spectrum disorder. Electrophoresis 35: 2046–2054. 10.1002/elps.201300370 24687421

[29] Feng C, Chen Y, Pan J, Yang A, Niu L, et al (2017) Redox proteomic identification of carbonylated proteins in autism plasma: insight into oxidative stress and its related biomarkers in autism. Clin Proteomics 14: 2 10.1186/s12014-017-9138-0 28077936 PMC5223466

[30] Cortelazzo A, De Felice C, Guerranti R, Signorini C, Leoncini S, et al (2016) Expression and oxidative modifications of plasma proteins in autism spectrum disorders: Interplay between inflammatory response and lipid peroxidation. Proteomics Clin Appl 10: 1103–1112. 10.1002/prca.201500076 27246309

[31] Shen L, Zhang K, Feng C, Chen Y, Li S, et al (2017) iTRAQ-Based Proteomic Analysis Reveals Protein Profile in Plasma from Children with Autism. Proteomics Clin Appl. 10.1002/prca.201700085 29274201

[32] Shen C, Zhao XL, Ju W, Zou XB, Huo LR, et al (2011) A proteomic investigation of B lymphocytes in an autistic family: a pilot study of exposure to natural rubber latex (NRL) may lead to autism. J Mol Neurosci 43: 443–452. 10.1007/s12031-010-9463-5 20957522

[33] Zerbo O, Yoshida C, Grether JK, Van de Water J, Ashwood P, et al (2014) Neonatal cytokines and chemokines and risk of Autism Spectrum Disorder: the Early Markers for Autism (EMA) study: a case-control study. J Neuroinflammation 11: 113 10.1186/1742-2094-11-113 24951035 PMC4080514

[34] Krakowiak P, Goines PE, Tancredi DJ, Ashwood P, Hansen RL, et al (2015) Neonatal Cytokine Profiles Associated with Autism Spectrum Disorder. Biol Psychiatry. 10.1016/j.biopsych.2015.08.007 PMC4753133 26392128

[35] Yang L, Rudser K (2016) Urine Protein Biomarker Candidates for Autism. J Proteomics Bioinform s14: 4.

[36] Ngounou Wetie AG, Wormwood KL, Charette L, Ryan JP, Woods AG, et al (2015) Comparative two-dimensional polyacrylamide gel electrophoresis of the salivary proteome of children with autism spectrum disorder. J Cell Mol Med 19: 2664–2678. 10.1111/jcmm.12658 26290361 PMC4627571

[37] Ngounou Wetie AG, Wormwood KL, Russell S, Ryan JP, Darie CC, et al (2015) A Pilot Proteomic Analysis of Salivary Biomarkers in Autism Spectrum Disorder. Autism Res 8: 338–350. 10.1002/aur.1450 25626423

[38] Castagnola M, Messana I, Inzitari R, Fanali C, Cabras T, et al (2008) Hypo-phosphorylation of salivary peptidome as a clue to the molecular pathogenesis of autism spectrum disorders. J Proteome Res 7: 5327–5332. 10.1021/pr8004088 19367726

[39] Hu VW (2012) Subphenotype-dependent disease markers for diagnosis and personalized treatment of autism spectrum disorders. Dis Markers 33: 277–288. 10.3233/DMA-2012-0916 22960334 PMC3810690

[40] Hu VW, Addington A, Hyman A (2011) Novel autism subtype-dependent genetic variants are revealed by quantitative trait and subphenotype association analyses of published GWAS data. PLoS One 6: e19067 10.1371/journal.pone.0019067 21556359 PMC3083416

[41] Hu VW, Steinberg ME (2009) Novel clustering of items from the Autism Diagnostic Interview-Revised to define phenotypes within autism spectrum disorders. Autism Res 2: 67–77. 10.1002/aur.72 19455643 PMC2737479

[42] Geschwind DH, Sowinski J, Lord C, Iversen P, Shestack J, et al (2001) The Autism Genetic Resource Exchange: A Resource for the Study of Autism and Related Neuropsychiatric Conditions. American Journal of Human Genetics 69: 463–466. 10.1086/321292 11452364 PMC1235320

[43] Barrett T, Troup DB, Wilhite SE, Ledoux P, Evangelista C, et al (2011) NCBI GEO: archive for functional genomics data sets—10 years on. Nucleic Acids Research 39: D1005–D1010. 10.1093/nar/gkq1184 21097893 PMC3013736

[44] Saeed AI, Sharov V, White J, Li J, Liang W, et al (2003) TM4: a free, open-source system for microarray data management and analysis. Biotechniques 34: 374–378. 10.2144/03342mt01 12613259

[45] Alter MD, Kharkar R, Ramsey KE, Craig DW, Melmed RD, et al (2011) Autism and increased paternal age related changes in global levels of gene expression regulation. PLoS One 6: e16715 10.1371/journal.pone.0016715 21379579 PMC3040743

[46] Gregg JP, Lit L, Baron CA, Hertz-Picciotto I, Walker W, et al (2008) Gene expression changes in children with autism. Genomics 91: 22–29. 10.1016/j.ygeno.2007.09.003 18006270

[47] Kong SW, Collins CD, Shimizu-Motohashi Y, Holm IA, Campbell MG, et al (2012) Characteristics and predictive value of blood transcriptome signature in males with autism spectrum disorders. PLoS One 7: e49475 10.1371/journal.pone.0049475 23227143 PMC3515554

[48] Pramparo T, Pierce K, Lombardo MV, Carter Barnes C, Marinero S, et al (2015) Prediction of autism by translation and immune/inflammation coexpressed genes in toddlers from pediatric community practices. JAMA Psychiatry 72: 386–394. 10.1001/jamapsychiatry.2014.3008 25739104

[49] Plaingam W, Sangsuthum S, Angkhasirisap W, Tencomnao T (2017) Kaempferia parviflora rhizome extract and Myristica fragrans volatile oil increase the levels of monoamine neurotransmitters and impact the proteomic profiles in the rat hippocampus: Mechanistic insights into their neuroprotective effects. Journal of Traditional and Complementary Medicine 7: 538–552. 10.1016/j.jtcme.2017.01.002 29034205 PMC5634759

[50] Perkins DN, Pappin DJ, Creasy DM, Cottrell JS (1999) Probability-based protein identification by searching sequence databases using mass spectrometry data. Electrophoresis 20: 3551–3567. 10.1002/(SICI)1522-2683(19991201)20:18<3551::AID-ELPS3551>3.0.CO;2-2 10612281

[51] Rebhan M, Chalifa-Caspi V, Prilusky J, Lancet D (1997) GeneCards: integrating information about genes, proteins and diseases. Trends Genet 13: 163 9097728 10.1016/s0168-9525(97)01103-7

[52] Xu LM, Li JR, Huang Y, Zhao M, Tang X, et al (2012) AutismKB: an evidence-based knowledgebase of autism genetics. Nucleic Acids Res 40: D1016–1022. 10.1093/nar/gkr1145 22139918 PMC3245106

[53] Li Q, Han Y, Dy ABC, Hagerman RJ (2017) The Gut Microbiota and Autism Spectrum Disorders. Front Cell Neurosci 11: 120 10.3389/fncel.2017.00120 28503135 PMC5408485

[54] Zoroglu SS, Armutcu F, Ozen S, Gurel A, Sivasli E, et al (2004) Increased oxidative stress and altered activities of erythrocyte free radical scavenging enzymes in autism. Eur Arch Psychiatry Clin Neurosci 254: 143–147. 10.1007/s00406-004-0456-7 15205966

[55] van Tilborg E, Achterberg EJM, van Kammen CM, van der Toorn A, Groenendaal F, et al (2018) Combined fetal inflammation and postnatal hypoxia causes myelin deficits and autism-like behavior in a rat model of diffuse white matter injury. Glia 66: 78–93. 10.1002/glia.23216 28925578 PMC5724703

[56] Szoko N, McShane AJ, Natowicz MR (2017) Proteomic explorations of autism spectrum disorder. Autism Res 10: 1460–1469. 10.1002/aur.1803 28509388

[57] Ujjainwala AL, Courtney CD, Rhoads SG, Rhodes JS, Christian CA (2017) Genetic loss of diazepam binding inhibitor in mice impairs social interest. Genes Brain Behav. 10.1111/gbb.12442 29193847

[58] Guidotti A, Forchetti CM, Corda MG, Konkel D, Bennett CD, et al (1983) Isolation, characterization, and purification to homogeneity of an endogenous polypeptide with agonistic action on benzodiazepine receptors. Proceedings of the National Academy of Sciences of the United States of America 80: 3531–3535. 6304714 10.1073/pnas.80.11.3531 PMC394079

[59] Alho H, Fremeau RT, Tiedge H, Wilcox J, Bovolin P, et al (1988) Diazepam binding inhibitor gene expression: location in brain and peripheral tissues of rat. Proceedings of the National Academy of Sciences of the United States of America 85: 7018–7022. 3413133 10.1073/pnas.85.18.7018 PMC282111

[60] Mori K, Emoto M, Inaba M (2011) Fetuin-A: a multifunctional protein. Recent Pat Endocr Metab Immune Drug Discov 5: 124–146. 22074587 10.2174/187221411799015372

[61] Elsas J, Sellhaus B, Herrmann M, Kinkeldey A, Weis J, et al (2013) Fetuin-a in the developing brain. Dev Neurobiol 73: 354–369. 10.1002/dneu.22064 23109215

[62] Allen-Brady K, Miller J, Matsunami N, Stevens J, Block H, et al (2009) A high-density SNP genome-wide linkage scan in a large autism extended pedigree. Mol Psychiatry 14: 590–600. 10.1038/mp.2008.14 18283277

[63] Breuss M, Heng JI, Poirier K, Tian G, Jaglin XH, et al (2012) Mutations in the beta-tubulin gene TUBB5 cause microcephaly with structural brain abnormalities. Cell Rep 2: 1554–1562. 10.1016/j.celrep.2012.11.017 23246003 PMC3595605

[64] Barbara M, Anna Livia L, Grazia G, Paolo C (2008) Autism and Metabolic Diseases. Journal of Child Neurology 23: 307–314. 10.1177/0883073807308698 18079313

[65] Rossignol DA, Frye RE (2012) Mitochondrial dysfunction in autism spectrum disorders: a systematic review and meta-analysis. Mol Psychiatry 17: 290–314. 10.1038/mp.2010.136 21263444 PMC3285768

[66] Hollis F, Kanellopoulos AK, Bagni C (2017) Mitochondrial dysfunction in Autism Spectrum Disorder: clinical features and perspectives. Curr Opin Neurobiol 45: 178–187. 10.1016/j.conb.2017.05.018 28628841

[67] Akbay EA, Moslehi J, Christensen CL, Saha S, Tchaicha JH, et al (2014) D-2-hydroxyglutarate produced by mutant IDH2 causes cardiomyopathy and neurodegeneration in mice. Genes Dev 28: 479–490. 10.1101/gad.231233.113 24589777 PMC3950345

[68] Stanfield AC, McIntosh AM, Spencer MD, Philip R, Gaur S, et al (2008) Towards a neuroanatomy of autism: a systematic review and meta-analysis of structural magnetic resonance imaging studies. Eur Psychiatry 23: 289–299. 10.1016/j.eurpsy.2007.05.006 17765485

[69] Chen R, Jiao Y, Herskovits EH (2011) Structural MRI in autism spectrum disorder. Pediatr Res 69: 63r–68r. 10.1203/PDR.0b013e318212c2b3 21289538 PMC3081653

[70] Brambilla P, Hardan A, di Nemi SU, Perez J, Soares JC, et al (2003) Brain anatomy and development in autism: review of structural MRI studies. Brain Res Bull 61: 557–569. 14519452 10.1016/j.brainresbull.2003.06.001

