# Supplementary figures and images for: Phenotypic subgrouping and multi-omics analyses reveal reduced diazepam-binding inhibitor (DBI) protein levels in autism spectrum disorder with severe language impairment
Source: PLoS One. 2019 Mar 28;14(3):e0214198. doi: 10.1371/journal.pone.0214198 (PMC6438570; doi:10.1371/journal.pone.0214198)

Control

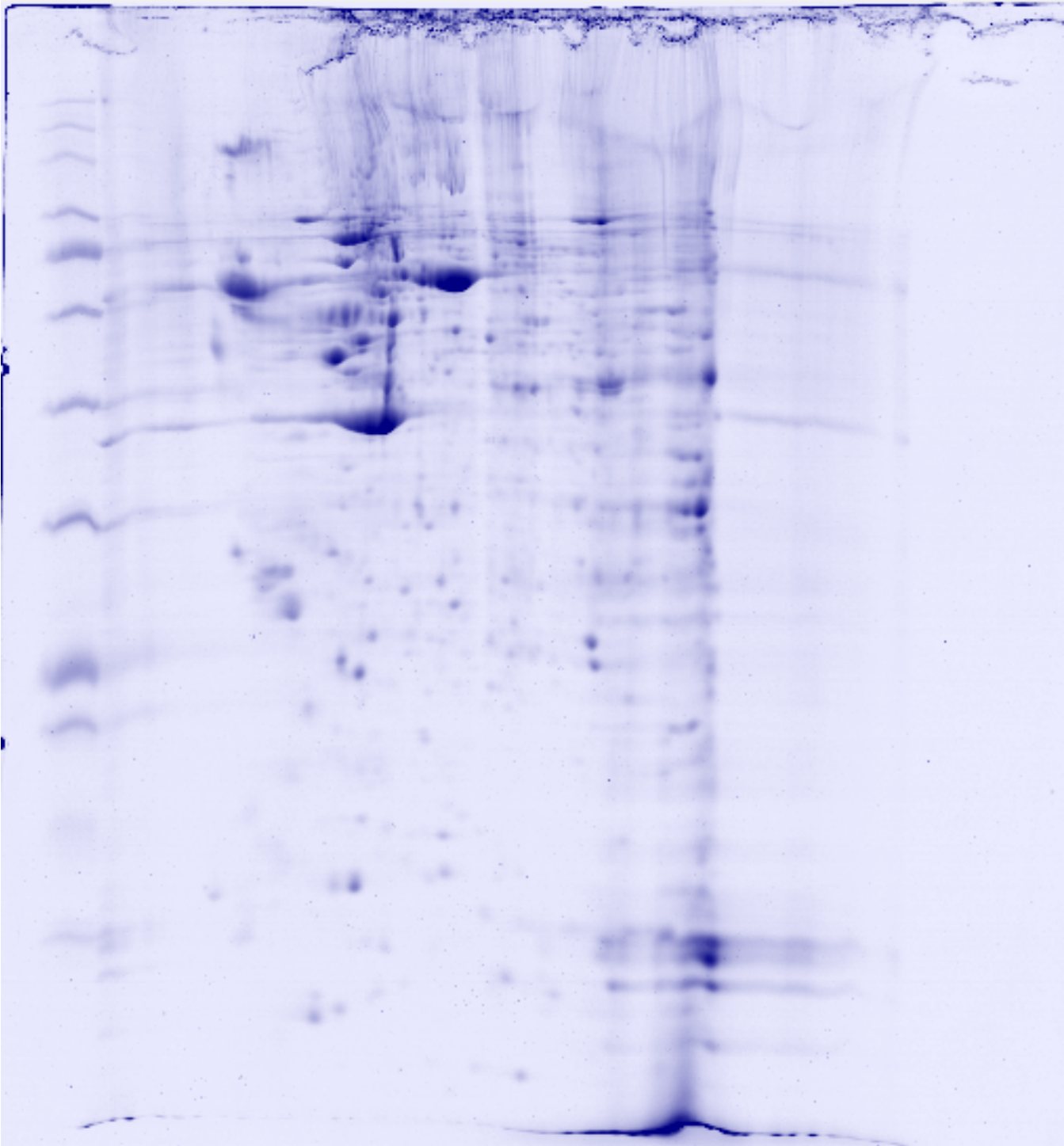

ASD\_G3 Red

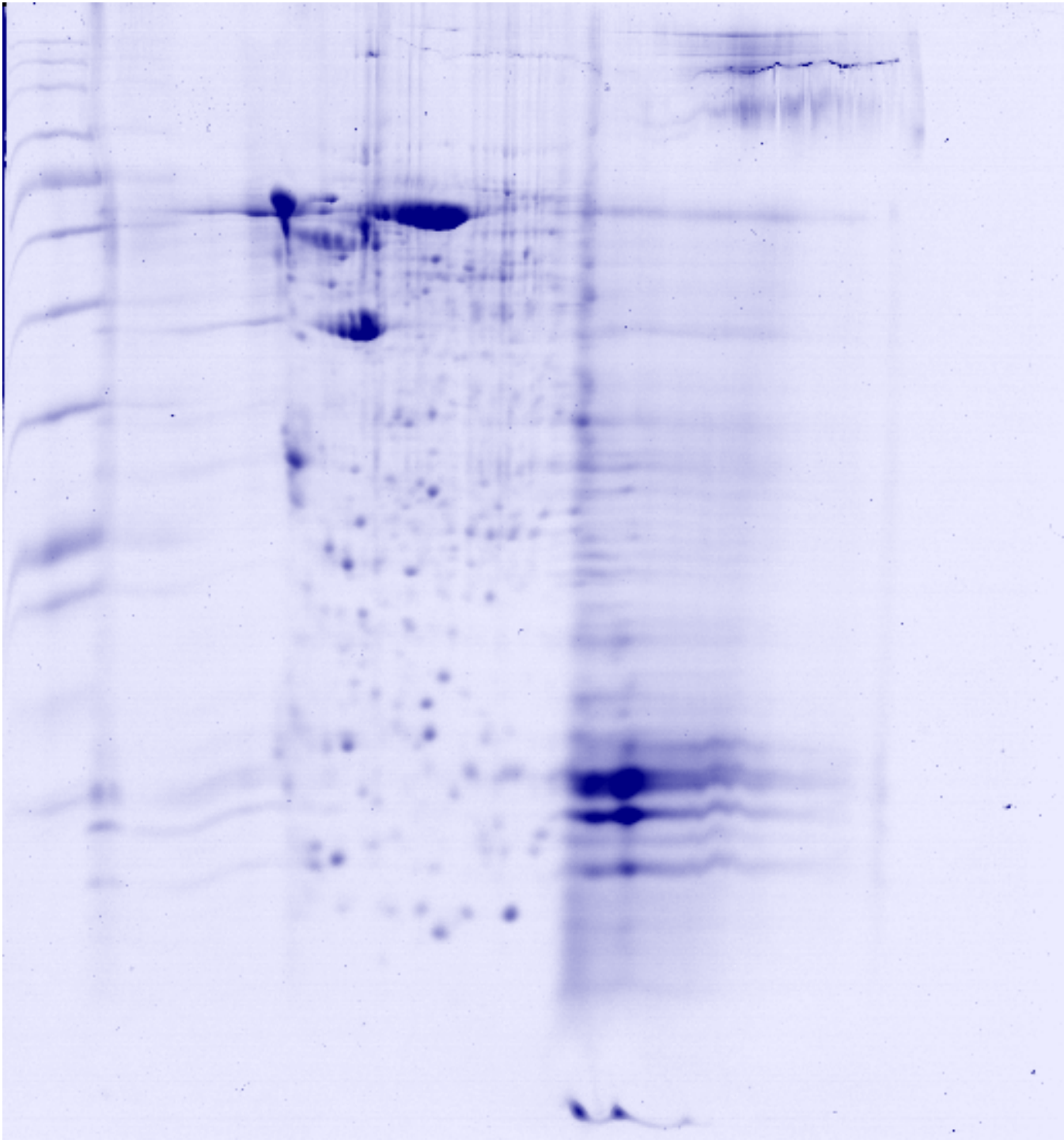

Supplement: S1 Fig — (PDF) [file pone.0214198.s007.pdf]

# Blue vs Control

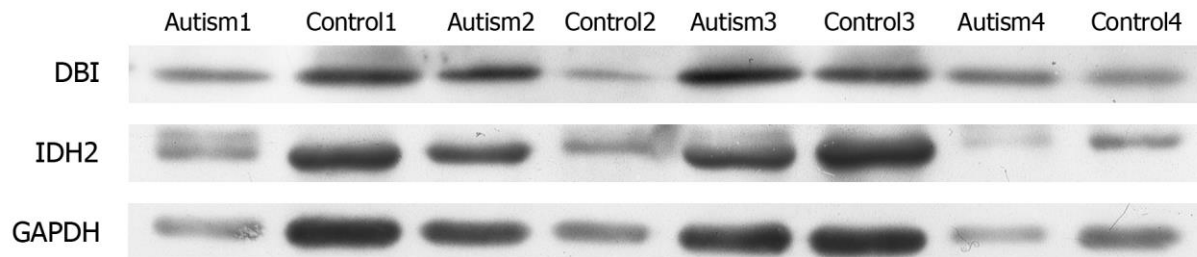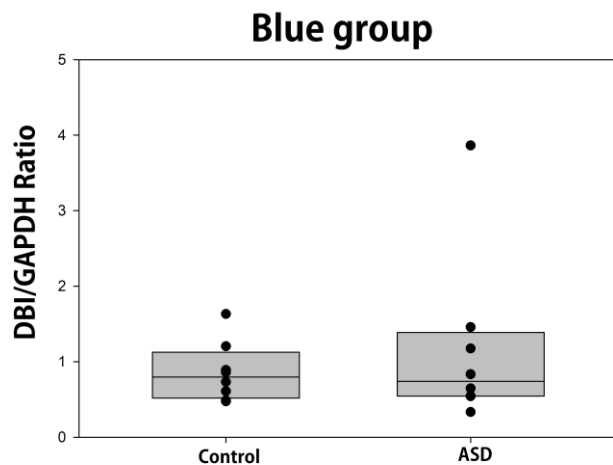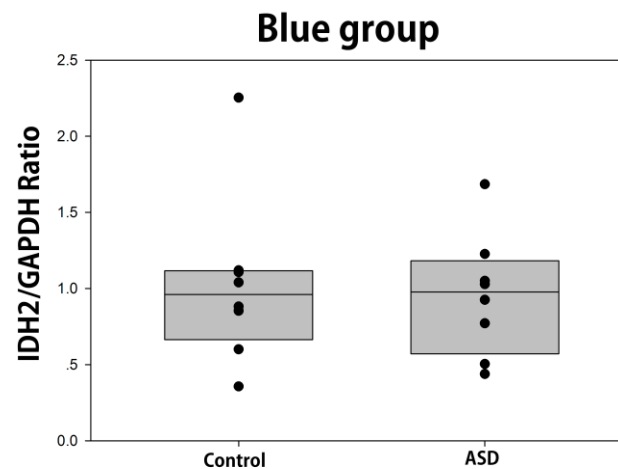

# Green vs Control

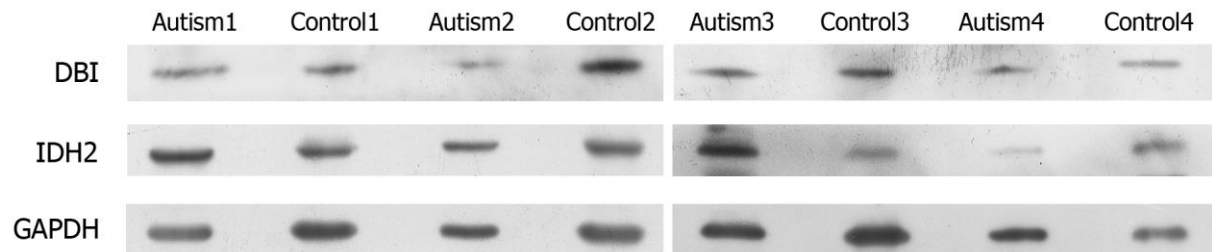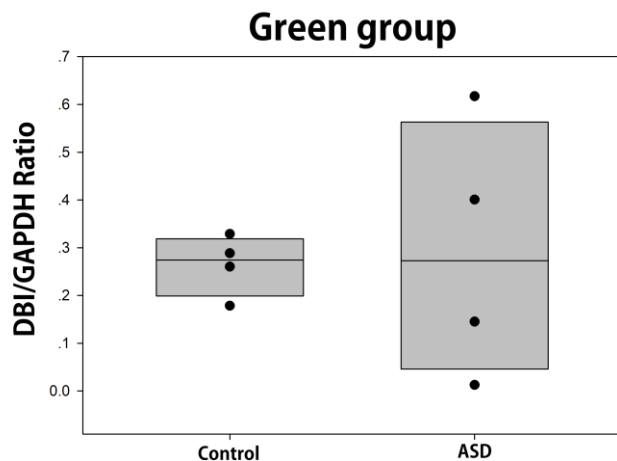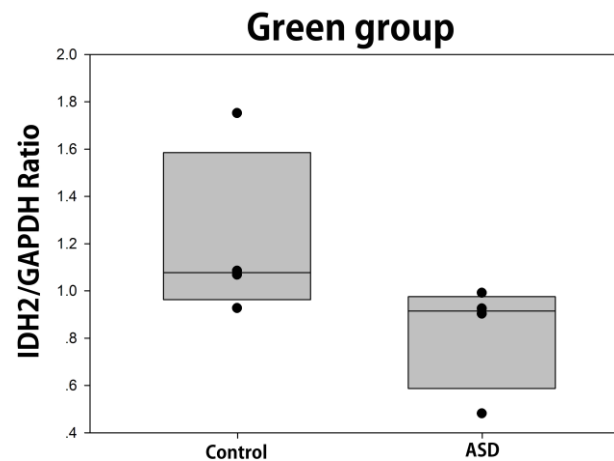

# Yellow vs Control

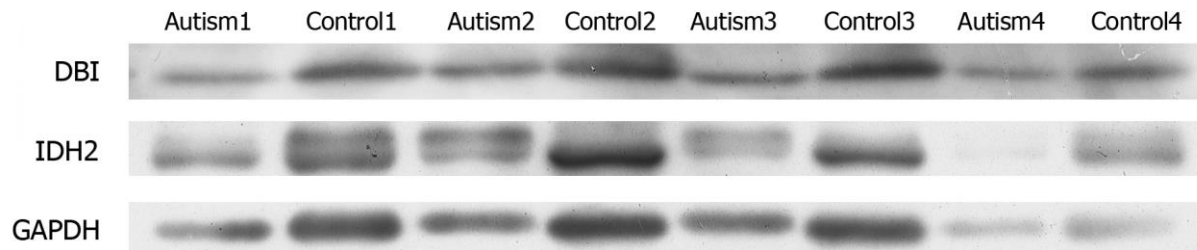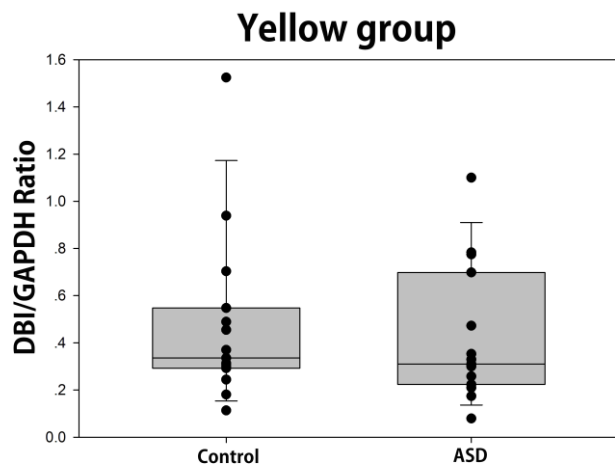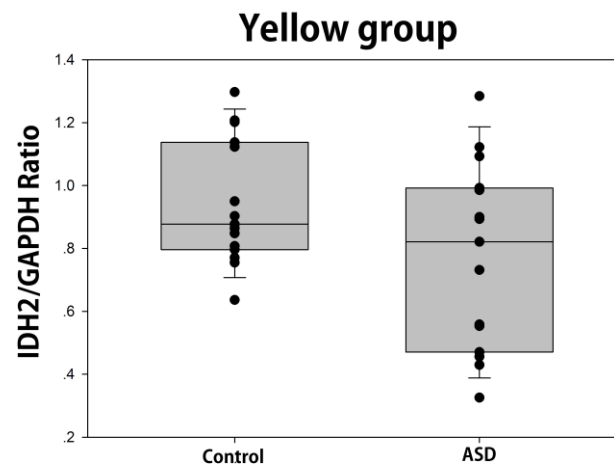

Supplement: S2 Fig — (PDF) [file pone.0214198.s008.pdf]
